# Impact of Time, Temperature, and Conformational Changes on the Characterization of Cardiotonic Steroid–Na^+^/K^+^-ATPase Interactions

**DOI:** 10.1007/s00232-026-00385-4

**Published:** 2026-07-27

**Authors:** Pedro Azalim-Neto, Leandro Barbosa, Cristoforo Scavone, Luis Eduardo M. Quintas

**Affiliations:** 1 Laboratório de Neurofarmacologia Molecular, Instituto de Ciências Biomédicas, Universidade de São Paulo, São Paulo, Brazil; 2 Laboratório de Bioquímica Celular, Universidade Federal de São João del- Rei (UFSJ) Campus Centro-Oeste Dona Lindu, Divinópolis, MG Brazil; 3 Laboratório de Farmacologia Bioquímica e Molecular, Instituto de Ciências Biomédicas, Universidade Federal do Rio de Janeiro, Rio de Janeiro, Brazil

**Keywords:** P-type ATPase modulation, Structure–function relationship, Thermodynamic profiling, High-throughput assays, Drug screening, Residence time.

## Abstract

Cardiotonic steroids (CTS) have gained prominence due to various mechanisms of action proposed or demonstrated to explain their effects, including their known classical and non-classical signaling pathways mediated by Na^+^/K^+^-ATPase (NKA) binding. For the characterization, interaction kinetics have been used to better translate in vivo efficacy and selectivity. In addition, temperature effects assessed by thermodynamic analysis provide deeper insight into intrinsic efficacy. However, the impact of conformational changes on both kinetic and thermodynamic parameters of CTS binding has been largely overlooked. This review discusses how these kinetic effects relate to efficacy and selectivity in the experimental characterization of CTS–NKA interactions. We focus on the most straightforward and high-throughput methods for describing CTS-NKA interaction, such as enzymatic activity inhibition, looking at dephosphorylation activity of NKA cycle, and compare them with gold-standard methods and its limitations. Finally, we include some statistical considerations for data analysis and future perspectives. This will provide better insight into the efficacy of this class of inhibitors/agonists targeting the NKA in drug discovery.

**Graphical Abstract:**

Drug-Target Interaction with Deep and Complex Binding Site.

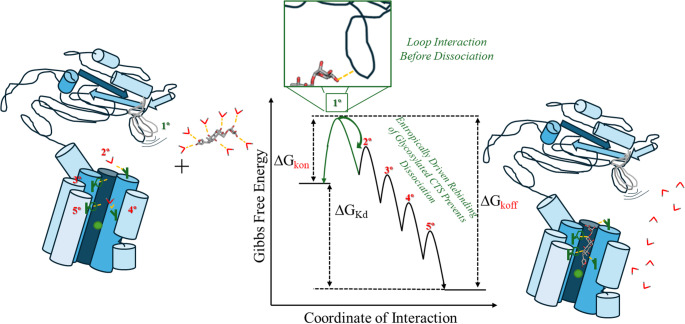

## Introduction

Cardiotonic steroids (CTS) returned to the spotlight due to numerous studies reporting their cytotoxic effects on cancer cells (Schneider et al. 2017; Kumavath et al. 2021; de Oliveira et al., 2021). Two important characteristics that impart these effects on cancer cells to CTS are the increased expression of Na⁺/K⁺-ATPase (NKA) isoforms (Sakai et al. 2004; Mijatovic et al. 2007; Toledo et al. 2021) and differences in the localization of NKA (Yang et al. 2014), depending on the type of cancer cell. This makes the interaction between CTS and NKA a promising approach for promoting sensitization to apoptosis, as well as for inhibiting the proliferation and migration of chemoresistant cancer cells (Mijatovic et al. 2012). Consequently, various mechanisms of action have been proposed or demonstrated to explain the antitumor effects of CTS, associated with their known canonical and non-canonical signaling pathways (Babula et al. 2013; Slingerland et al. 2013). In this line, some studies have shown that modification of the osidic portion has increased the affinity to NKA and increased cell cycle arrest in A549 and H460 cell lines (Schneider et al. 2018). On the other hand, modification of the lactone ring that decreases the affinity for NKA has kept the cytotoxicity effect on HeLa and RKO (Rocha et al. 2014), reducing its proliferation by inhibition of the EGFR/ERK signaling pathway (Pessôa et al. 2020) and the inflammatory reaction produced by carrageenan in murine models (Vieira et al. 2018). Thus, these findings highlight how such chemical alterations can serve as a powerful tool for cancer and inflammation research.

The characterization of ligands that focuses on the rate of interaction has been used to better translate in vitro results into in vivo efficacy and selectivity, affecting not only the pharmacodynamics but also the pharmacokinetic/pharmacodynamic profile of molecules (Copeland 2021). Additionally, the effect of temperature, evaluated through thermodynamic analysis, is a key approach that provides a deeper description of the intrinsic efficacy (Borea et al. 2000) and selectivity (Tarcsay and Keserű 2015) of the ligand via its interaction energy. However, the initial characterization of CTS requires particular attention, as NKA is a protein that undergoes multiple conformational changes during its catalytic cycle to perform both phosphorylation and dephosphorylation, drastically altering its binding site. The understanding of conformational effects on both kinetic and thermodynamic parameters of CTS interaction has been largely overlooked.

This review highlights the importance of understanding the kinetics, thermodynamics and conformational effects of NKA on the of CTS, with the aim of better elucidating their link to efficacy and selectivity in the characterization of CTS-NKA interactions. We focus on the simplest and most high-throughput methods for characterizing CTS-NKA interactions, including enzymatic activity inhibition, looking at dephosphorylation activity of NKA cycle and its limitations. Finally, we include some statistical considerations for data analysis and future perspectives. This will provide better insight into the efficacy of this class of inhibitors/ligands targeting the Na⁺/K⁺ pump in drug discovery.

## Kinetics of Drug-Target Interaction

Since the enzyme–inhibitor interaction in the reaction medium can be described by the Clark model (Clark 1933; Maehle 2005), the potency of inhibition will be directly correlated with binding affinity, as signal amplification is absent in this type of experiment, and the interaction follows the law of mass action. A simple one-step model can initially describe this inhibition:

$$ \:D + R\underset{{k_{{off}} }}{\overset{{k_{{on}} }}{\rightleftharpoons}}\:DR $$

Where D and R represent the drug and receptor concentration, respectively, *k*_on_ represents the second-order rate of association (M^− 1^.s^− 1^), and *k*_off_ represents a first-order rate of dissociation (s^− 1^). Working with an equilibrium endpoint, which is more common in the CTS–NKA literature, the data can be fitted with a hyperbolic function, such as the equation proposed by Archibald V. Hill (Hill 1910; Maehle 2005),

1 $$\:B=\frac{{B}_{max}*{D}^{h}}{{{K}_{d}}^{h}+{D}^{h}}$$

Where D represents the concentration of the drug (M unit), *K*_d_ represents the equilibrium dissociation constant (M unit), and *h* represents the Hill coefficient (dimensionless value). B and B_max_ represent the bound ligand and the maximum binding capacity, respectively. However, their units are determined by the experimental method and may vary across assays. The binding of ECT to NKA is traditionally measured by radiolabeled binding assays. In this context, B and B_max_ are typically expressed as a concentration relative to the amount of protein, most commonly in fmol/mg protein or pmol/mg protein.

Instead of equilibrium assays, it is more informative to determine the rate of interaction that can be achieved using a concentration–response progression curve. In this case, when time is considered in the experiment, the data follows a one-phase exponential association:

2 $$\:{B}_{x}={B}_{max}\frac{D}{{K}_{d}+D}\left(1-{e}^{-\left({k}_{on}\left[D\right]+{k}_{off}\right)x}\right)$$

Where B_x_ is the amount of bound ligand at a time point x, and consequently, the rate parameters will be on the x^− 1^ scale. If, in the graph of concentration response progression curve, the x-axis is plotted in minutes (as many CTS respond in a time of minutes to hours), the *k*_off_ unit will be min^− 1^, and *k*_on_ will be M^− 1^.min^− 1^. For using the standard unit of respective rate of reaction, that will be s^− 1^, is recommended to transform as $\:{k}_{off}=\frac{x}{60}\:{s}^{-1}$ and $\:{k}_{on}=\frac{x}{60}\:{M}^{-1}.{s}^{-1}$, where x is the value of the respective rate obtained. It is also possible to insert the transformations into the progression curve equation, substituting e^− 1^ to e^− 60^. The same can be done if the x-axis is plotted in hours (in this case, 3,600 s). As a substitute for using *K*_d_, it is possible to use the *k*_off_/*k*_on_ ratio. In this case, only two parameters are adjusted to fit the entire progression curve across different concentrations (a global fitting), and fixing B_max_ also facilitates fitting the full curve using this equation.

Considering that potency of inhibition will be directly correlated with binding affinity, Normalizing the active inhibition at different inhibitor concentrations against the total activity in the absence of inhibitor (yielding a percentage of inhibition, Eq. 3) allows for the use of the same models described for binding kinetics (Eq. 2).

3 $$\:Inb\:=100-\left(\frac{Ab{s}_{m}-Ab{s}_{b}}{Ab{s}_{t}-Ab{s}_{b}}\:100\right)$$

Where Abs represents the absorbance measured to determine the molecule concentration (m), basal (b) activity, and total (t) activity, respectively.

This approach is beneficial for the initial characterization of both equilibrium and rate constants of binding/inhibition. Equation 2 is available in GraphPad Prism (GraphPad Software, Boston, MA, USA; www.graphpad.com) as “*two or more concentrations of hotnm*”. In this equation on Prism, the concentration is entered in nM, but the unit of *K*_d_ is in M. The x-axis is on a minute time scale; consequently, *k*_off_ is min^− 1^ and *k*_on_ is M^− 1^. min^− 1^. For a good fit, both the inflection of the curve (described by the rates) and its plateau (described by occupancy) need to be captured.

Since NKA exhibits both phosphorylation and dephosphorylation activities, kinetic determination of the former is, in many cases, limited and often requires a coupled enzymatic system (ATP regeneration via NADH) for real-time monitoring of NKA activity (Radnai et al. 2019). For the latter activity, however, experiments can be readily performed using the substrate *para*-nitrophenyl phosphate (pNPP). This enzymatic method produces *para*-nitrophenol (pNP) as a product, which exhibits a yellow color, allowing spectrophotometric detection at neutral to alkaline pH, enabling automated reading during the incubation period, as well as the use of 96-well plates, combining high-throughput with high-performance screening (Azalim-Neto et al. 2024).

**Fig. 1:**
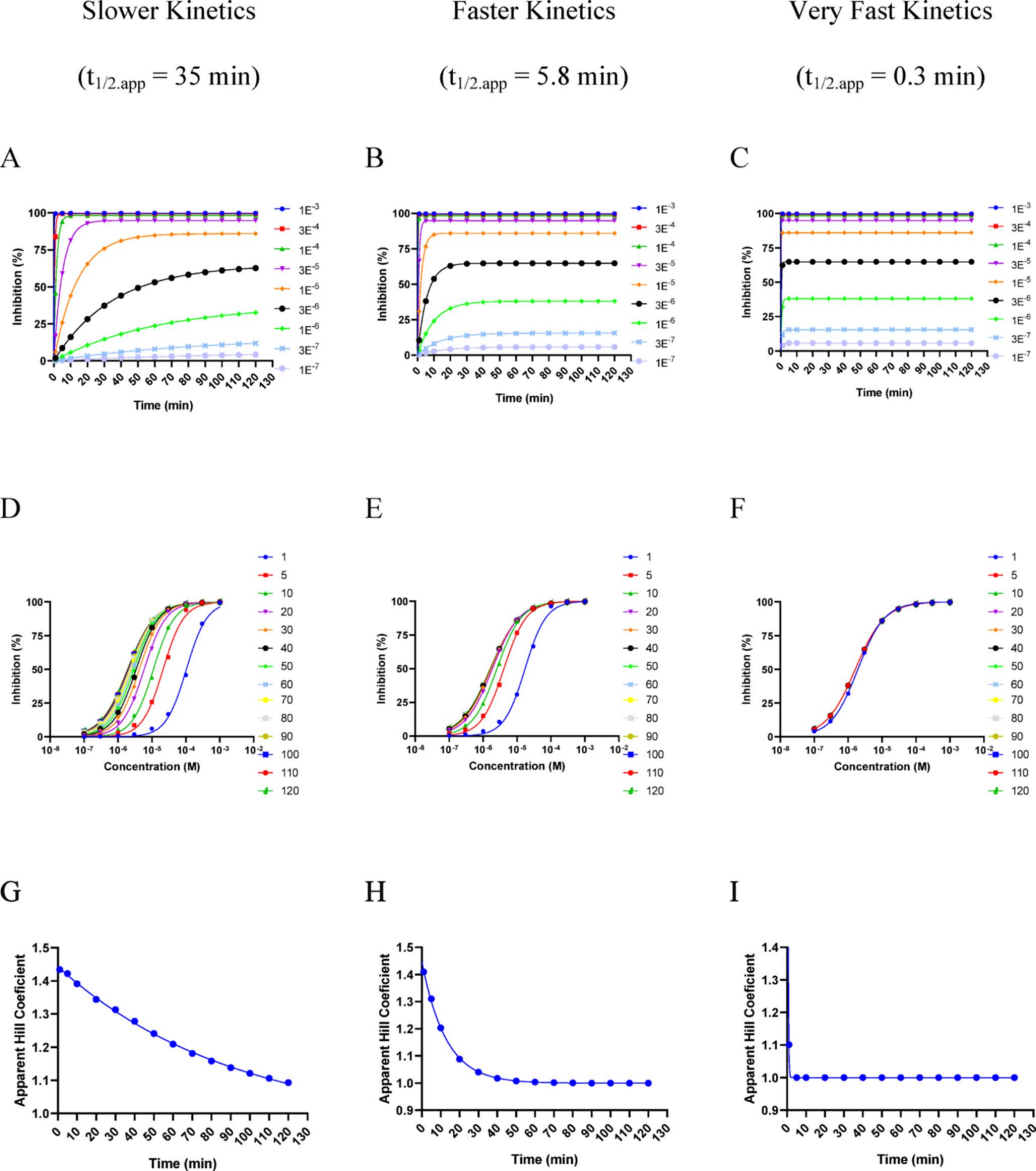
Simulated data of a slower, faster, and very fast kinetics molecule (classification relative to each *k*_obs_ = *k*_on_ × *K*_d_ + *k*_off_) with the equilibrium constant (1.6 × 10^− 6^ M). In **A**, **B**, and **C**, concentration-response progression curve data fitting with three different molecules showing different values of *k*_on_ and *k*_off_. In **D**, **E**, and **F**, concentration-response curves of different times of respective molecules; **G**, **H**, and **I** are the exponential decay of the apparent Hill coefficient over time of respective molecules. The data were generated with the rate values presented in Table 1, using Eq. 2, with a concentration range from 10^− 7^ to 10^− 3^ M, and B_max_ was fixed at 100. The concentration-response curve was fitted with Eq. 1, with B_max_ fixed at 100. A high concentration was included to ensure accurate determination of the maximal inhibition of each concentration

Identifying only the equilibrium binding or inhibition constant does not adequately describe the profile of a molecule. By examining the time-dependent progression of binding or inhibition, it becomes evident that specific structural modifications can lead a molecule to reach the same affinity value through distinct kinetic pathways. Figure 1; Table 1 illustrate a scenario in which three different molecules attain the same equilibrium value but exhibit different apparent affinities and, consequently, may erroneously display Hill coefficients different from 1, not due to a cooperative mechanism, but rather because equilibrium was not properly established at the appropriate time point for these molecules. The time required to reach apparent equilibrium can be assessed by determining *k*_obs_= *k*_on_[*K*_d.app_] + *k*_off_ and by t_1/2.app_ = 0.693/*k*_obs_.

**Table 1:** Values of the equilibrium and rate constants, along with their respective energetic parameters, used to generate the plots for the three molecules that exhibit the same affinity and free energy at 37 °C but display distinct kinetic and energetic composition profiles

Molecule	1	2	3
ΔH^‡^_kon_ (kJ.mol^− 1^)	30.0	50.1	24.0
ΔS^‡^_kon_ (J.K^− 1^.mol^− 1^)	-110	-30.0	-90.0
ΔG^‡^_kon_ (kJ.mol^− 1^)	64.1	59.4	51.9
k_on_ (M^− 1^.s^− 1^)	103	638	1.2 × 10^+ 4^
ΔH^‡^_koff_ (kJ.mol^− 1^)	-2.00	54.7	64.6
ΔS^‡^_koff_ (J.K^− 1^.mol^− 1^)	-324	-126	-70.0
ΔG^‡^_koff_ (kJ.mol^− 1^)	98.5	93.8	86.3
K_off_ (s^− 1^)	1.7 × 10^− 4^	1.0 × 10^− 3^	1.9 × 10^− 2^
τ (s)	5.9 × 10^+ 3^	1.0 × 10^+ 3^	52.3
t_1/2,diss_ (s)	4.1 × 10^+ 3^	693	34.5
ΔH_*Ka*_ (kJ.mol^− 1^)	32.0	-4.60	-40.6
ΔS_*Ka*_ (J.K^− 1^.mol^− 1^)	214	96.0	-20.0
ΔG_*Ka*_ (kJ.mol^− 1^)	-34.4	-34.4	-34.4
*K*_*d*_ (M)	1.6 × 10^− 6^	1.6 × 10^− 6^	1.6 × 10^− 6^
k_obs.eq−app_ (s^− 1^)	3.3 × 10^− 4^	2.0 × 10^− 3^	3.8 × 10^− 2^
t_1/2.eq−app_ (min)	35.0	5.80	0.30

Consequently, considering these simulations, one of the major challenges in CTS–NKA interaction characterization is, for example, the incubation time, as some studies have not accounted for the time-dependent inhibition characteristics of certain CTS. These studies often spend very short incubation times, thereby underestimating affinity since equilibrium is not reached (Noël 2017). This can also lead some authors to speculate about elevated Hill coefficients, although for incorrect reasons (Vauquelin 2016). As *k*_on_ is a second-order rate ($\:v={k}_{on}\left[D\right]\left[R\right]$), the higher the concentration, the faster the plateau will be reached. This is particularly relevant when working with short incubation times due to the stability of the protein. However, *k*_off_ is a first-order rate ($\:v={k}_{off}\left[DR\right]$) and, therefore, is not affected by concentration, but rather depends on the strength or complexity of the interaction. Thus, molecules with very slow dissociation rates may not be properly characterized over short time scales, even with increased concentrations (which are typically limited experimentally by the aqueous solubility of the compound). The *k*_off_ is more related to the strength of interaction and can define the time a molecule spends bonded on the receptor, for example, using the concept of residence time, τ (s), that is, the inverse of *k*_off_ (1/*k*_off_) or by the dissociation half-time, t_1/2.diss_ (s), (0.693/*k*_off_) (Copeland 2021).

### Thermodynamics of Drug-Target Interaction

Given this context, the key aspect of this characterization is to determine which energetic components govern both the equilibrium and the kinetics of these molecules. The fundamental parameter in thermochemistry was described by J. Willard Gibbs (Gibbs 1876–1878), as it describes the spontaneity of reactions, the Gibbs free energy (Atkins and De Paula 2017). It is characterized by both the potential energy contained in molecules to do useful work at constant pressure (the enthalpy), as well as the energy spent on the kinetic energy of molecules that dictates the direction of the reaction, known as entropy:

4 $$\:\varDelta\:G^{o}=\:\varDelta\:H^{o}-T\varDelta\:S^{o}$$

Where ∆G° (J.mol^− 1^) is the Gibbs free energy change, ∆H° (J.mol^− 1^) is the enthalpy change, ∆S° (J.K^− 1^.mol^− 1^) is the entropy change, and T is the absolute temperature in Kelvin (K). In this way, when the reactants (or protein and ligand) show a negative change in Gibbs free energy from the initial to the final state, this indicates that the reaction favors the formation of products (or the complex). By this equation, the spontaneity (negative increase in ∆G°) can be achieved by a negative increase in the enthalpy change, or a positive increase in the entropic component (T∆S°).

Enthalpy in drug-target interactions is primarily governed by the strengths of hydrogen bonding, ionic interactions, and van der Waals forces, with contributions from hydrophobic effects and π-π stacking. Entropy, on the other hand, is governed by the amount of energy spent on kinetics, such as vibration, translocation, and rotation of the solvent, molecule, or protein residue. While a favorable enthalpy (negative one) is directly affected by the potential energy of the strength of interaction, entropy, on the other hand, will be favorable (positive) when kinetic energy is increased, or will be unfavorable (negative) when kinetic energy is restricted. As temperature is a macroscopic measure proportional to the average kinetic energy of molecular motion in a system, the higher the temperature, the higher the entropic component.

**Fig. 2:**
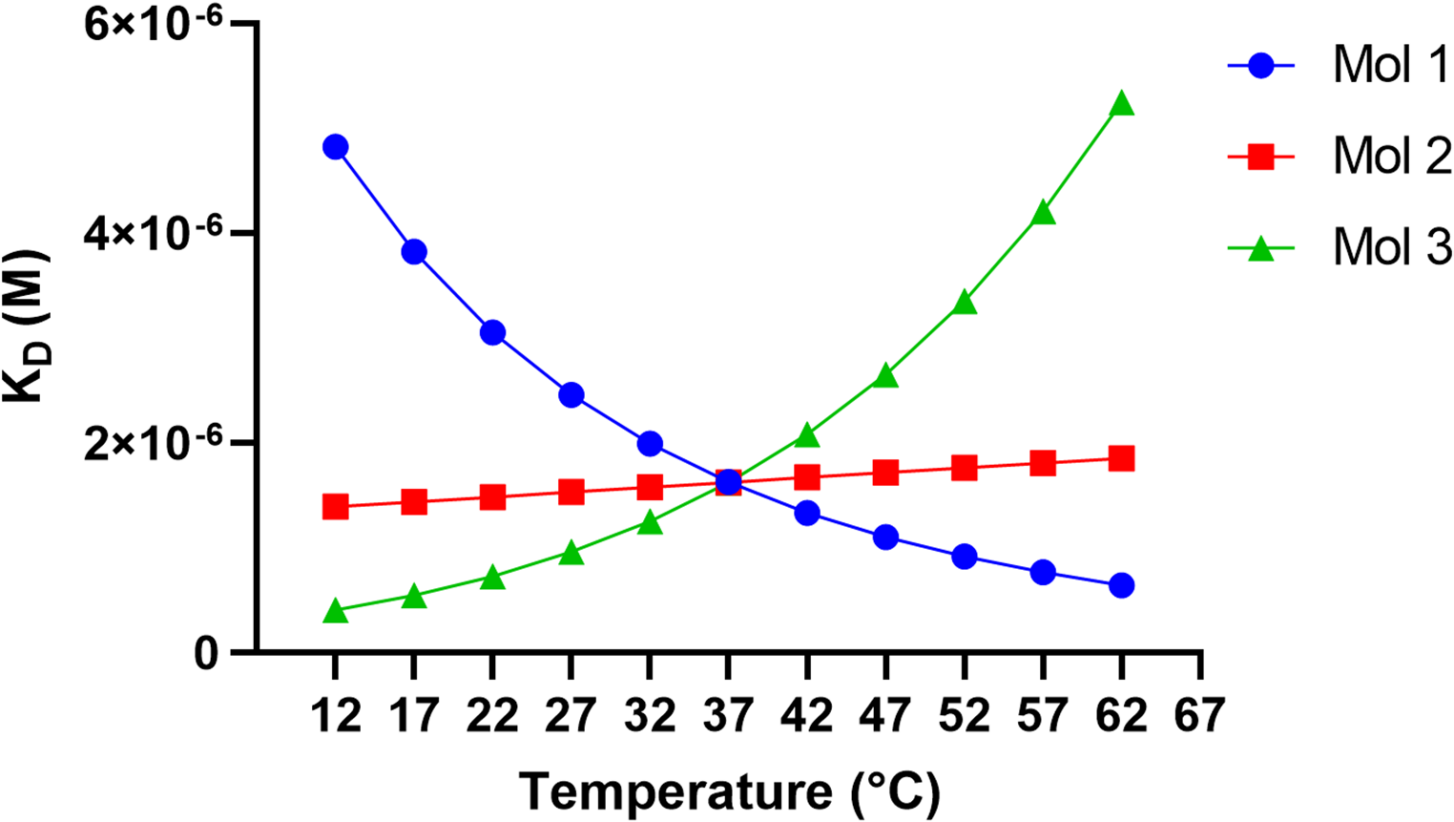
Simulated data for three molecules (Mol 1, Mol 2, and Mol 3) exhibiting the same affinity at 37 °C. The graph shows the relationship between the equilibrium dissociation constant (K_d_, in molar units) and temperature in degrees Celsius. These data were generated using the linear Van’t Hoff equation (Eq. 5), based on the ΔH and ΔS values reported in Table 1

To illustrate the importance of thermodynamics in structure–activity relationships (SAR), consider a scenario in which, during the characterization of the same three molecules, all are found to exhibit the same affinity (1.6 × 10^− 6^ M) at 37 °C, leading to the erroneous conclusion that the chemical modifications produce equivalent effects. However, upon evaluating their affinities at different temperatures, it becomes evident that these molecules reach the same affinity at 37 °C through distinct thermodynamic pathways (Fig. 2).

As shown in Fig. 2, Mol 1 exhibits increased affinity with rising temperature, whereas Mol 2 is unaffected by temperature, and Mol 3 shows a decrease in affinity as temperature increases. Based on this profile, the objective is now to characterize the energetic contributions that govern each of these three molecules.

The fundamental equation that links thermodynamic quantities with the composition of a system at equilibrium (in pharmacology, *K*_a_, the inverse of *K*_d_) was described by Jacobus H. Van’t Hoff (Van’t Hoff 1884), when analyzing the effect of temperature on the equilibrium constant.

5 $$\:\varDelta\:{G}^{o}=\:-RT\:\mathrm{l}\mathrm{n}\mathrm{K}$$

The Van’t Hoff equation will be presented in its dimensional linear model:

6 $$\:\mathrm{ln}\left(K\right)=-\frac{\varDelta\:H^{o}}{R}\cdot\:\frac{1}{T}+\frac{\varDelta\:S^{o}}{R}$$

However, in this model, one important point must be considered: it cannot take dimensional values as units, only dimensionless values. *K* (which, in this case, corresponds to *K*_a_) is expressed in M^− 1^; this is corrected by multiplying it by the standard concentration (C^°^ = 1 M):

7 $$\:\mathrm{ln}\left({K}_{a}{C}^{o}\right)=-\frac{\varDelta\:H^{o}}{R}\cdot\:\frac{1}{T}+\frac{\varDelta\:S^{o}}{R}$$

In the graph of $\:\mathrm{ln}\left({K}_{\mathrm{a}}{C}^{o}\right)$ against $\:\frac{1}{T}$, that should be linear if the heat capacity is close to 0, the slope is $\:-\frac{\varDelta\:H^{o}}{R}$ and the intercept is $\:\frac{\varDelta\:S^{o}}{R}$, consequently the $\:\varDelta\:H^{o}$ and $\:\varDelta\:S^{o}$ can be determined by multiplying by -R and R, respectively.    

**Fig. 3:**
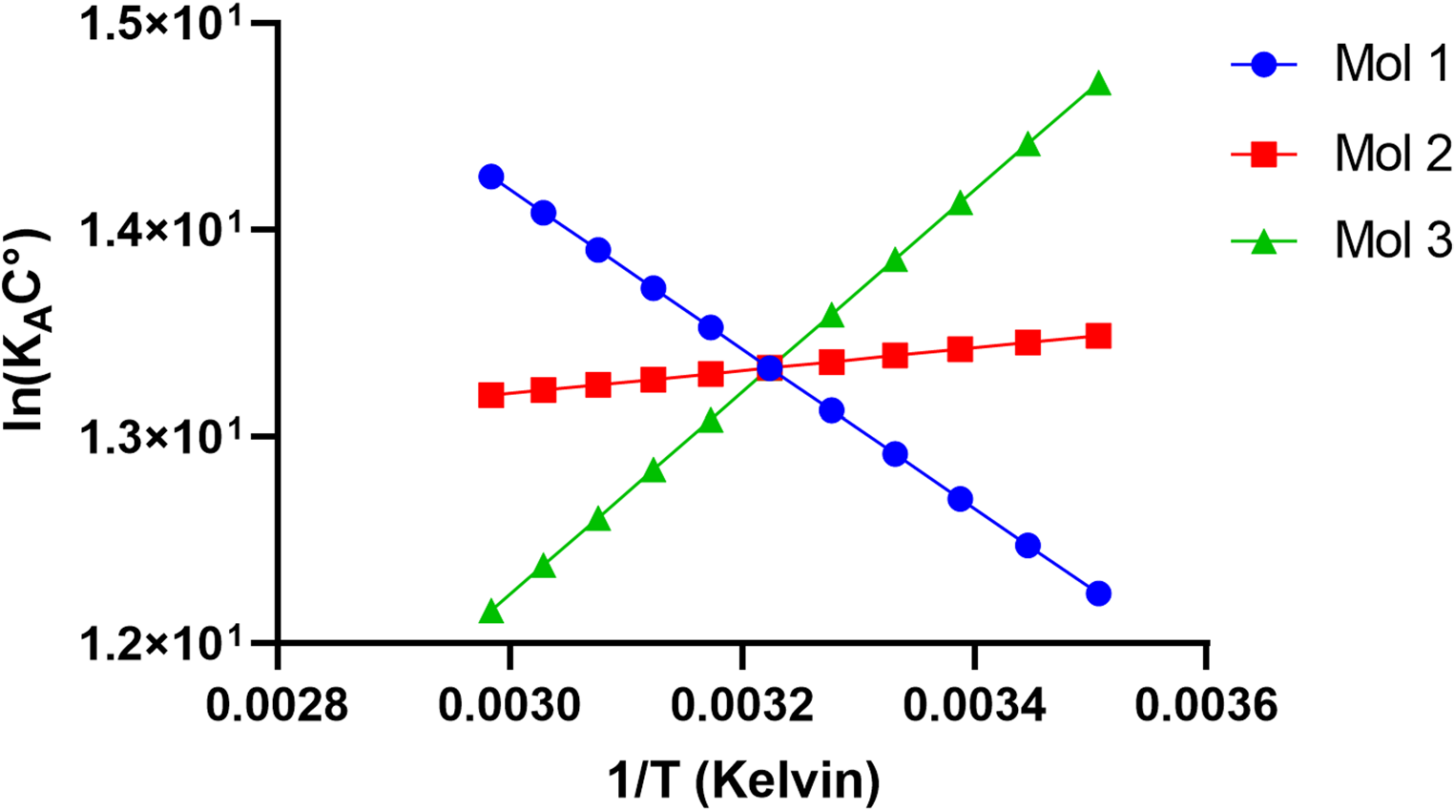
Van’t Hoff plot for three molecules (Mol 1, Mol 2, and Mol 3) with the same affinity at 37 °C. The graph shows the relationship between the natural logarithm of the equilibrium association constant and the inverse of temperature (in Kelvin). These data were generated using the linear Van’t Hoff equation, based on the values reported in Table 1

Using the linear form of the Van’t Hoff equation (Fig. 3), it was possible to determine that the affinity of Mol 1 is strongly driven by an entropic gain (214 J.K^− 1^.mol^− 1^), offset by an enthalpic penalty (32 kJ.mol^− 1^), whereas Mol 2 is largely unaffected by temperature due to its favorable entropy (96 J.K^− 1^.mol^− 1^), but without an enthalpy penalty (-4.6 kJ.mol^− 1^). Mol 3, in contrast, displays affinity predominantly driven by enthalpy (-40.6 kJ.mol^− 1^), accompanied by an entropic penalty (-20 J.K^− 1^.mol^− 1^).

**Fig. 4:**
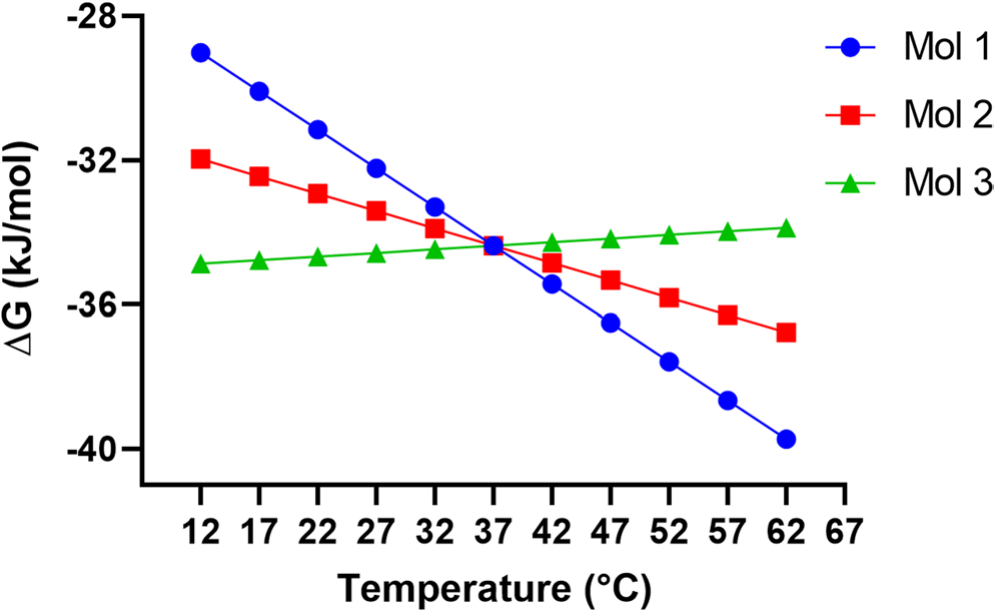
Free energy of binding for three different molecules (Mol 1, Mol 2, and Mol 3). The graph shows the binding free energy as a function of temperature (in degrees Celsius) for three molecules with distinct energetic profiles. These data were generated using the Gibbs free energy equation, based on the values reported in Table 1

Finally, Fig. 4 displays the energetic profile of each molecule in terms of ΔG: two are entropy-driven (ΔG becomes more negative with increasing temperature), albeit to different extents, while the third is enthalpy-driven.

**Fig. 5:**
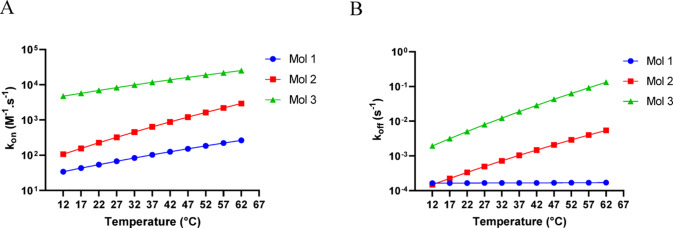
Rate constants (power of 10) at different temperatures for three molecules (Mol 1, Mol 2, and Mol 3). In (**A**), the association rate constant is shown as a function of temperature (in degrees Celsius). In (**B**), the dissociation rate constant is presented across different temperatures. These data were generated using the Eyring equation, based on the values reported in Table 1

Recognizing that the equilibrium constant is defined by the ratio of the rate constants, one can examine how the rate constants of each molecule behave independently across a range of temperatures (Fig. 5).

By the effect of temperature on the rate of reaction, Svante Arrhenius (Arrhenius 1887) describes an energy barrier that governs the rate of product (or complex) formation:

8 $$\:k=A{e}^{\left(\frac{{-E}_{A}}{RT}\right)}$$

Where A is the Arrhenius constant, E_A_ is the energy of activation that represents the energy necessary to overcome the barrier that determines the rate of reaction.

Finally, Henry Eyring, Meredith Gwynne Evans, and Michael Polanyi associated the description of Arrhenius with the thermodynamic quantities and described the component of this barrier with the concept of the transition state (Eyring 1935; Evans and Polanyi 1935). In this theoretical state, the interaction is at high energy, which needs to be overcome for the formation of the product (or complex ground state):

9 $$\:k=\frac{{k}_{B}T}{h}{e}^{\frac{-\varDelta\:G^{\ddagger}}{RT}}$$

Where k is the rate of reaction (in the context of protein-ligand, the rate of interaction), *k*_B_ is the Boltzmann constant (1.380649 × 10⁻²³ J/K), and *h* is the Planck constant (6.62607015 × 10⁻³⁴ J·s). Deriving the Gibbs free energy, the Eyring equation is complemented with the enthalpic and entropic description:

10 $$\:k=\frac{{k}_{B}T}{h}{e}^{\frac{{\varDelta\:S}^{\ddagger}}{R}}{e}^{\frac{-{\varDelta\:H}^{\ddagger}}{RT}}$$

The Eyring equation will frequently be presented in its dimensional linear model:

11 $$\:\mathrm{ln}\left(\frac{k}{T}\right)=-\frac{{\varDelta\:H}^{\ddagger}}{R}\cdot\:\frac{1}{T}+\left(\frac{{\varDelta\:S}^{\ddagger}}{R}+\mathrm{ln}\left(\:\frac{{k}_{B}}{h}\right)\right)$$

However, two important points should be considered in this model. First, logarithmic functions formally require dimensionless arguments. Thus, expressions such as $\:\mathrm{l}\mathrm{n}({k}_{\mathrm{o}\mathrm{f}\mathrm{f}}/T)$are not rigorously dimensionless if $\:{k}_{\mathrm{o}\mathrm{f}\mathrm{f}}\:$is expressed in $\:{s}^{-1}$. Nevertheless, in chemical kinetics, this treatment is commonly accepted under the implicit assumption of normalization by standard reference units. Second, the ratio $\:{k}_{B}/h$has units of $\:{K}^{-1}{s}^{-1}$, since $\:{k}_{\mathrm{B}}\:$and $\:h$ have units of $\:J{K}^{-1}$and $\:Js$, respectively. When multiplied by temperature, the resulting term $\:\left({k}_{B}T/h\right)$ correctly assumes units of $\:{s}^{-1}$, consistent with a first-order rate constant such as $\:{k}_{\mathrm{o}\mathrm{f}\mathrm{f}}$.

However, in this model, we have two points that need to be appreciated: as before, *ln* cannot receive dimensional values with units, only dimensionless values. For *k*_off_, that is s^− 1^, we will have a k s^− 1^/T K^− 1^; consequently, *ln* receives a dimensional value. However, both terms will cancel each other (s^− 1^/K^− 1^), resulting in a dimensionless value. Also, *k*_B_ and *h* have units, J.K^− 1^ and J.s, respectively, resulting in a term that does not completely cancel each other (resulting in K^− 1^.s^− 1^). The model that has been applied to use both terms on a dimensionless value for *k*_off_ is:

12 $$\:\mathrm{ln}\left(\frac{{k}_{off}}{{k}_{B}T/h}\right)=-\frac{{\varDelta\:H}^{\ddagger}}{R}\cdot\:\frac{1}{T}+\frac{{\varDelta\:S}^{\ddagger}}{R}$$

In this way, the *k*_B_T/*h* now cancel each other, leaving s^− 1^, which will be cancelled by *k*_off_, which is also s^− 1^.

For *k*_on_, however, that is a second-order rate of M^− 1^.s^− 1^; this treatment still has a dimensional value of M^− 1^. To cancel the unit, we multiply by a standard concentration (C^°^= 1 M):

13 $$\:\mathrm{ln}\left(\frac{{k}_{on}{C}^{^\circ\:}}{{k}_{B}T/h}\right)=-\frac{{\varDelta\:H}^{\ddagger}}{R}\cdot\:\frac{1}{T}+\frac{{\varDelta\:S}^{\ddagger}}{R}$$

In the graph of $\:\mathrm{ln}\left(\frac{k}{{k}_{B}T/h}\right)$ against $\:\frac{1}{T}$, the slope is $\:-\frac{{\varDelta\:H}^{\ddagger}}{R}$ and the intercept is $\:\frac{{\varDelta\:S}^{\ddagger}}{R}$, consequently the $\:{\varDelta\:H}^{\ddagger}$ and $\:{\varDelta\:S}^{\ddagger}$ can be determined by multiplying by -R and R, respectively.

While the affinity is determined by the energy difference between the final bound state and the initial unbound state, the rate constants are determined by the energy barrier between states. The composition of this energy barrier can be described by Eyring transition state theory. In this way, the energy difference between the unbound state and the transition state can explain the association rate, *k*_on,_ and the energy between the bound state and the transition state describes the dissociation rate, *k*_off_.

Furthermore, as the equilibrium of the protein–ligand interaction is governed by these two rate constants, subtracting the enthalpy, entropy, and Gibbs free energy of the association rate from the respective properties of the dissociation rate provides the thermodynamic parameters of the equilibrium, as described by the Van’t Hoff equation.

**Fig. 6:**
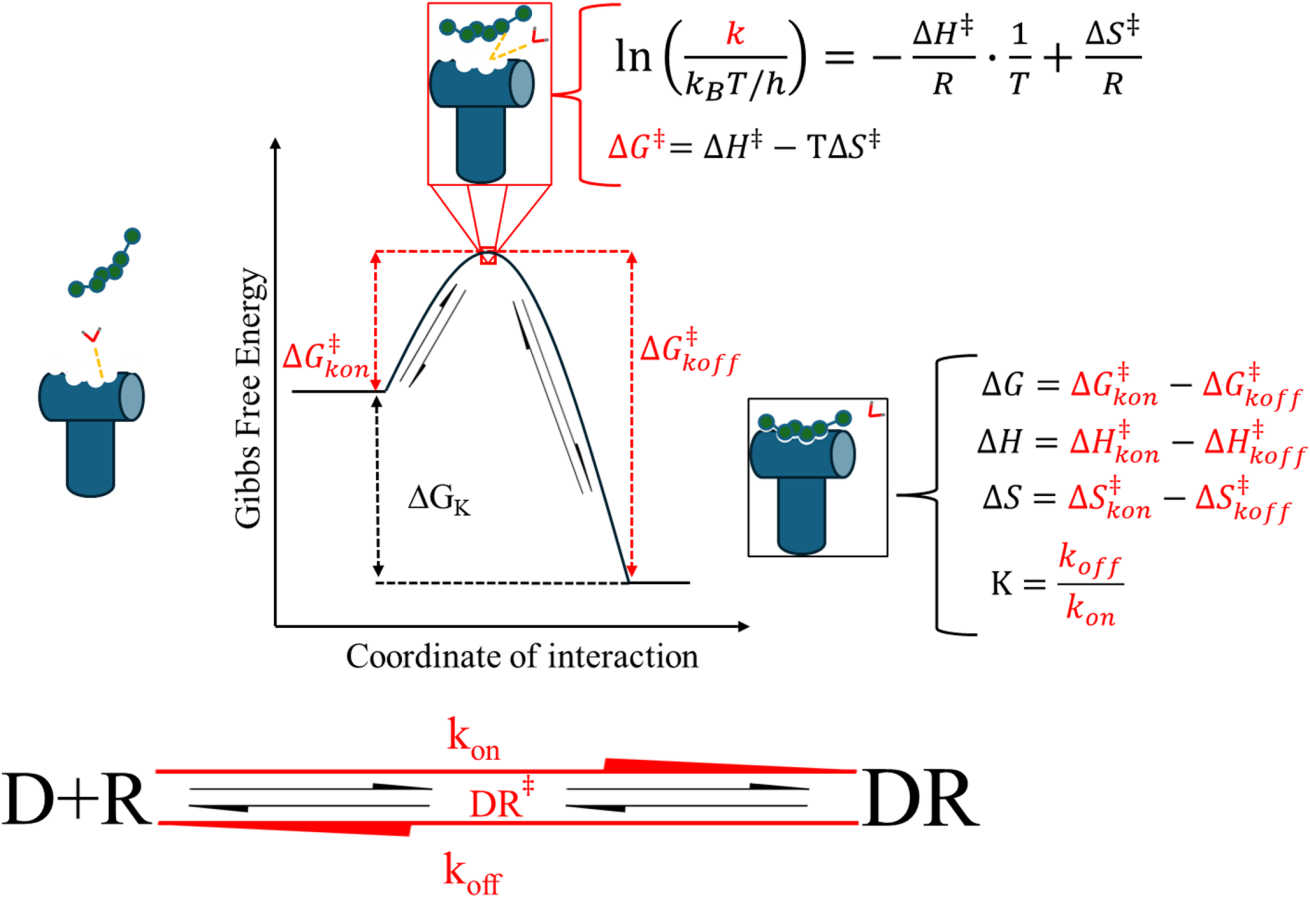
The image illustrates the free energy that governs the coordination of the drug with a surface receptor. The rate of interaction between the drug (green) and the receptor (blue) will be determined by the molecule with enough energy necessary to overcome the transition state, which can be, for example, the desolvation of a water molecule in the main residue of the binding site, to finally reach the ground state, which forms the ground state interaction. A faster molecule is the one that finds a coordinate with a low energy necessary for the transition state (can be enthalpically or entropically dominated), that can culminate in the same equilibrium affinity/potency, but different rate. At the end, the difference in the energy of association and dissociation results in the energy of the equilibrium ground state

In the context of Eyring transition state theory, analyzing rate constants, the free energy change of the transition state (ΔG^‡^) is always positive, as it indicates the energy required to overcome the transition barrier, either to reach the bound state (in the case of association) or the unbound state (in the case of dissociation). The enthalpy changes of the transition state (ΔH^‡^) are also typically positive, as they represent the energy required to break and form bonds to surpass the transition state. The entropy changes of the transition state (ΔS^‡^) can be either negative, meaning the transition state has fewer microstates and is therefore less probable (often described as more rigid or less favorable), or positive, indicating a greater number of microstates and a more probable (more flexible or favorable) transition (Fig. 6).

**Fig. 7:**
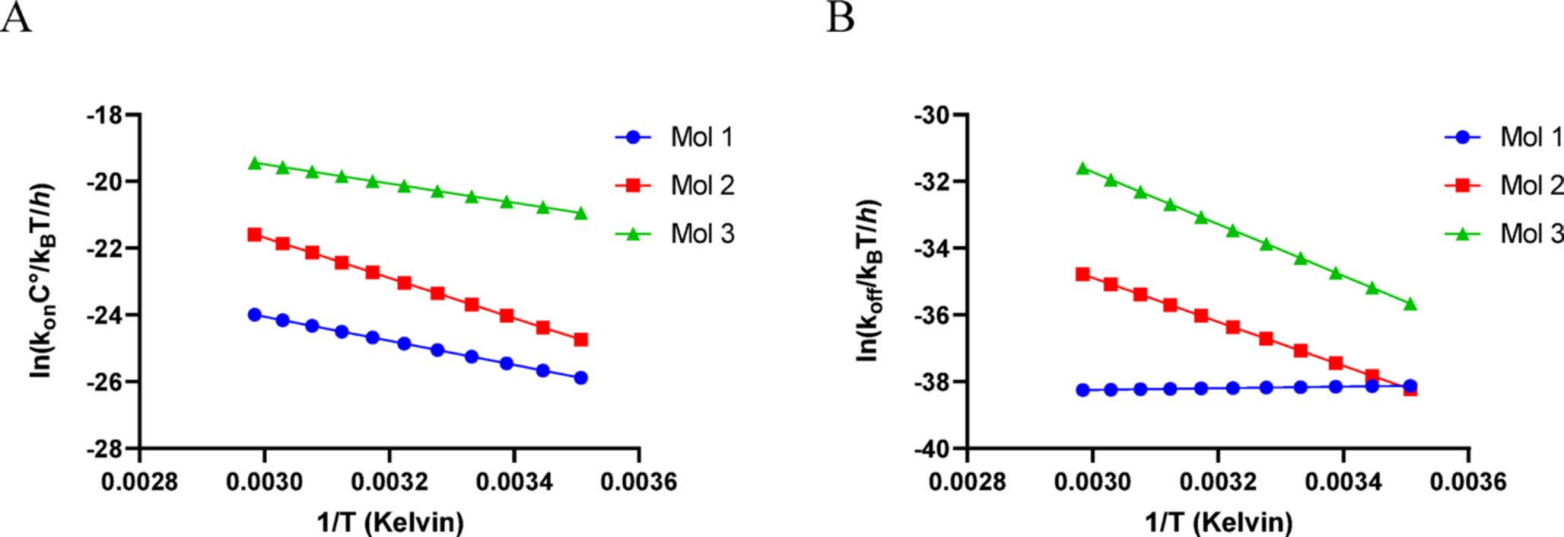
Eyring plot. In (**A**), the natural logarithm of the association rate constant over by temperature is plotted against the inverse of temperature for three molecules (Mol 1, Mol 2, and Mol 3) with the same affinity at 37 °C. In (**B**), the natural logarithm of the dissociation rate constant normalized by temperature (ln(k/T)) is shown as a function of the inverse temperature for the three molecules

**Fig. 8:**
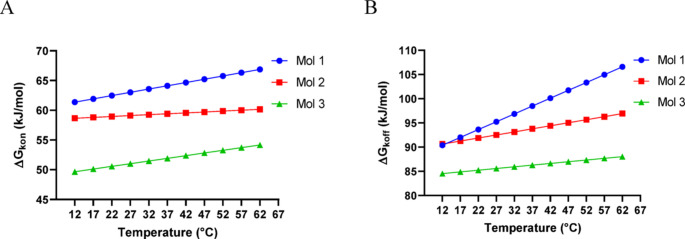
Transition-state energy barriers of the rate constants. In (**A**), the Gibbs free energy of activation for the association process is shown as a function of temperature (in degrees Celsius) for three molecules (Mol 1, Mol 2, and Mol 3) with the same affinity at 37 °C. In (**B**), the Gibbs free energy of activation for the dissociation process is presented as a function of temperature for the three molecules

Using the linear form of the Eyring equation, it was possible to determine, for example, that the Mol 1 exhibits slow equilibration due to a high energetic barrier for the association process (ΔG^‡^_*k*on_ = 64.1 kJ.mol^− 1^) relative to its counterparts (Fig. 7). However, at equilibrium, the interaction remains energetically favorable (ΔG_*Ka*_ *=* -34.4 kJ.mol^− 1^), comparable to the other molecules, as the complexity of the interaction leads to very slow dissociation due to the higher energy barrier (ΔG^‡^_*k*off_ = 98.5 kJ.mol^− 1^; Fig. 8). This is supported by the negative enthalpy and large entropy contributions, indicating a protein–ligand complex that is highly unfavorable to dissociate. As a result, increasing temperature does not increase the dissociation rate, in contrast to its counterparts, or may, in part, reduce the dissociation rate with increasing temperature due to the complex dissociation.

Similarly, this also highlights the issue associated with using inappropriate incubation conditions. Also, it is not uncommon in published papers for researchers to follow the reagent kit instructions, often conducting experiments at room temperature (25 °C). Under such conditions, the interaction of the studied compound may not be accurately identified. Given that protein–ligand interactions can exhibit different kinetic profiles, it becomes important to evaluate which thermodynamic component governs these phenomena; for instance, an entropically driven CTS will be very dependent on temperature (Azalim-Neto et al. 2024).

## Structure-Activity Relationship on CTS-NKA Interaction

**Fig. 9:**
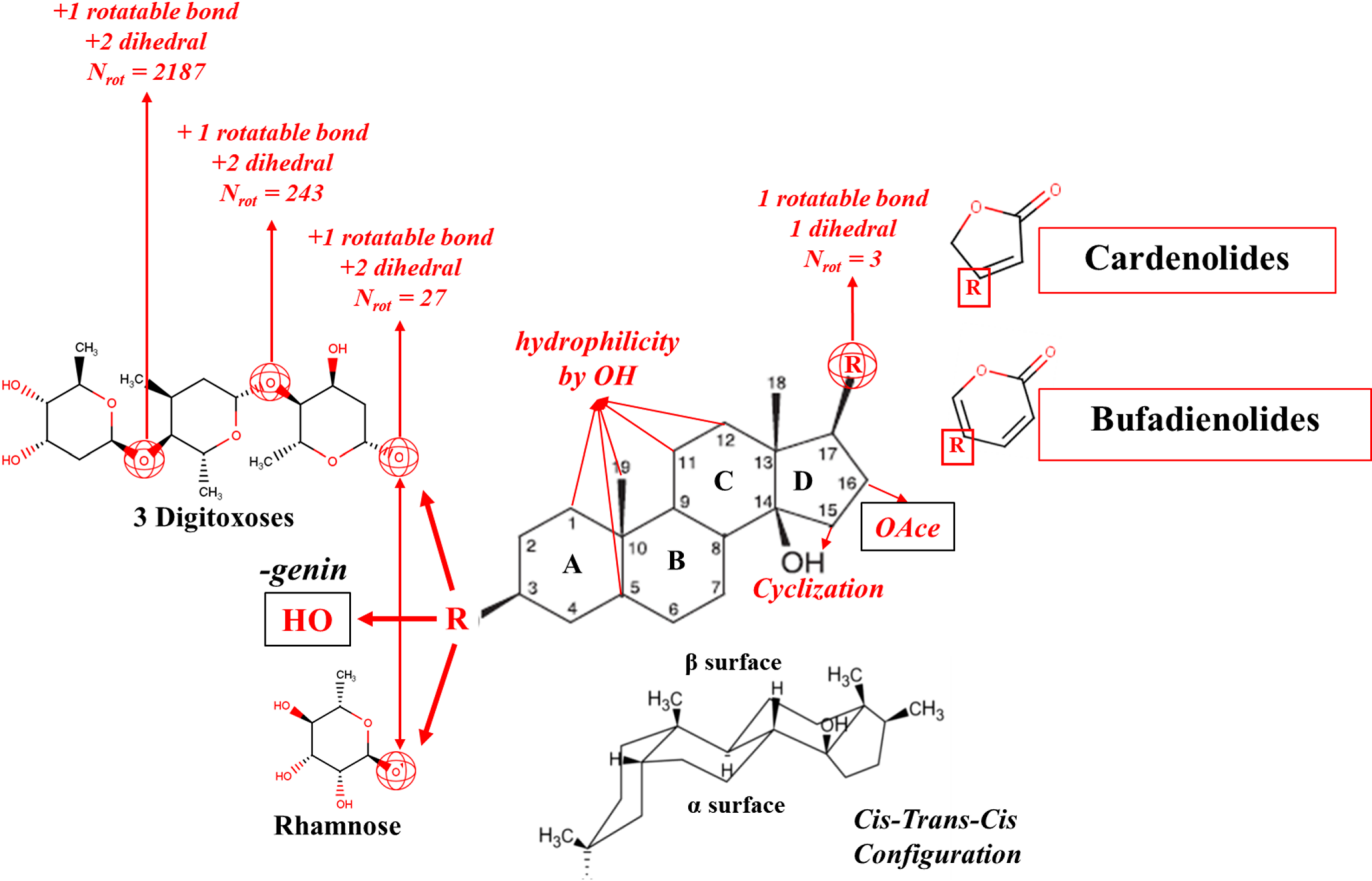
– Structure of naturally obtained cardiotonic steroids and marked in red all common modifications from the preserved steroid nucleus. In the center, the steroidal nucleus and the numbering of its atoms. Below the nucleus is the representation of its three-dimensional configuration. On the left are the possible substitutions at carbon C3, usually a glycosidic moiety, ranging from a single rhamnose (e.g., ouabain) to three digitoxoses (e.g., digitoxin) naturally occurring, as well as the absence of a glycosidic portion characterized by the presence of a hydroxyl group, leading to the suffix -genin in this cardiotonic steroid (for example, ouabagenin or digitoxigenin). On the right are the substitutions at carbon C17 that define the class: a five-membered ring in the case of cardenolides or a six-membered ring in the case of bufadienolides. The flexibility of some CTS is marked with the mesh sphere and expressed as the number of theoretical rotamers (N_rot_), considering 3 theoretical states

CTS are composed of three main regions that guide this interaction (Fig. 9). The first is the steroidal core, with *cis* A–B, *trans* B–C, and *cis* C–D ring junctions, which form a chair-like conformation (El-Seedi et al. 2019). The second region is the lactone ring with partial charge distribution at C17 of the D ring, which defines the class of CTS: either cardenolides (with a five-membered butenolide ring) or bufadienolides (with a six-membered α-pyrone ring). The third main structural region is at the C3 position of the steroid nucleus, normally a glycosylation. When no conjugated group is present, a hydroxyl group occupies this position, and such compounds are classified with the suffix **-**genin (El-Seedi et al. 2019).

**Fig. 10:**
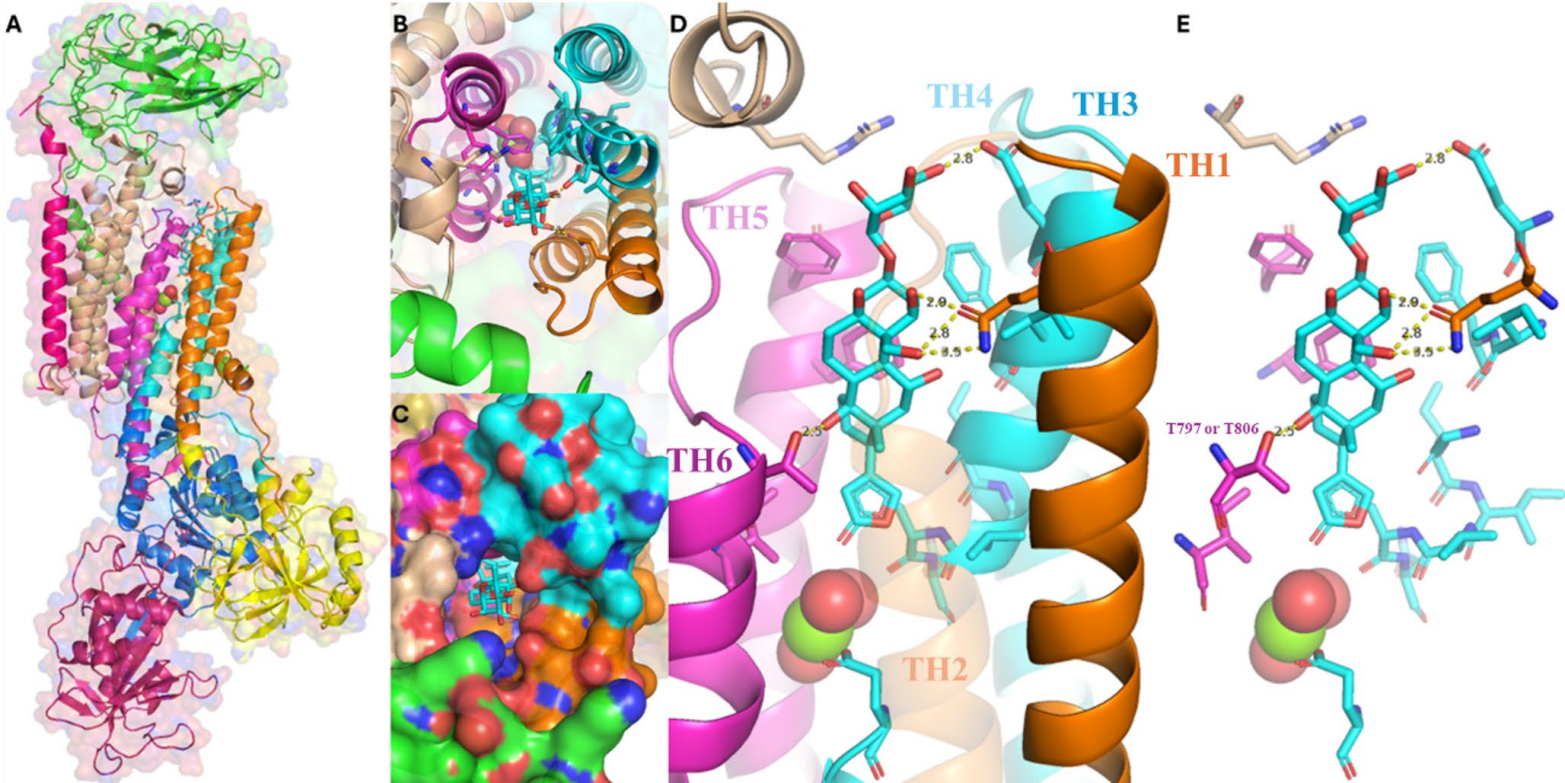
Crystallographic structure of the Na^+^/K^+^-ATPase in the E2P conformation (PDB ID: 7WZ0 from *Squalus acanthias*) bound to ouabain is shown. In A, the illustration highlights the α-subunit, composed of the transmembrane helix αTH1–2 (orange), αTH3–4 (light blue), αTH5–6 (purple), and αTH7–8 (brown), as well as the P domain (phosphorylation domain, dark blue), the A domain (actuator domain, yellow), and the N domain (nucleotide-binding domain, red). The β-subunit is depicted in green, and the FXYD subunit in light pink. In B and C, view of the extracellular cavity from the β-subunit as a cartoon and solvent accessible surface, respectively. In D, view of the binding site of ouabain from αTH2 (transparency) with the close (3.5 A) H-bound distance (yellow dashes). In E, only the residues surrounding ouabain (αTH2 was hiding)

The NKA is a heterotrimeric enzyme complex comprising α, β, and γ (FXYD2) subunits, with the α and β subunits essential for its catalytic activity. The α-subunit, which constitutes the catalytic core, comprises more than 1,010 amino acid residues (~ 110 kDa) organized into 10 transmembrane helices. It contains binding sites for sodium, potassium, magnesium ions, and CTS on the transmembrane helix, and ATP on the cytoplasmic domain (Ogawa et al., 2009, 2015; Laursen et al. 2013, 2015; Kanai et al. 2020). CTS access their binding site from the extracellular side, specifically in conformations where the binding pocket is exposed to the extracellular environment. The extracellular pocket is approximately 25 Å in height and is formed by the first six transmembrane helices (αTH1–6). At the base of this pocket, within the central region of the transmembrane helix, lie the ion-binding sites I and II, which can be occupied by potassium, sodium, or magnesium ions. The CTS binding site is located between ion-binding site II and the entrance of the extracellular pocket, spanning the remaining ~ 20 Å of the pocket. CTS are typically bound when one of these ions occupies the ion-binding sites, stabilizing conformations that are competent for CTS binding (Laursen et al. 2013, 2015; Kanai et al. 2020; Fig. 10).

## Steroidal Core

The binding mode of CTS is highly conserved across multiple crystallographic structures reported in the literature. In contrast to more superficial binding sites, binding energy and affinity are not determined exclusively by the ground state; consequently, binding dynamics play a significant and increasingly relevant role in determining molecular affinity. In CTS, a hydroxyl group at C14 on the α-face is responsible for a hydrophilic interaction with threonine on TM6, while the β-face is oriented toward TM1–2 (Ogawa et al., 2009, 2015; Laursen et al. 2013, 2015; Kanai et al. 2020).

Most of the enthalpic contribution or entropic penalty in CTS arises from substitutions on the steroidal core and, consequently, from their interactions, whether hydrophilic or hydrophobic, with the αTH1–4 for beta surface or αTH5-6 for α surface. Although desolvation is primarily an entropically driven process (and normally will produce an enthalpy penalty), as part of the energy will be spent on the kinetic energy of water molecules, it can also be enthalpically favorable when it involves the removal of solvent molecules from hydrophobic moieties, such as methyl groups, rather than from polar groups like hydroxyls. Desolvation becomes energetically advantageous only if the hydroxyl group engages in strong compensatory interactions within the binding interface (Harper and Black 2007; Lafont et al. 2007).

Because this is a deep binding site, the rate and ability of a ligand to penetrate the binding pocket may be inversely proportional to the number of hydrogen bond donors and acceptors present in the steroidal core. Strong interactions formed during the association process can slow progression toward the ground state. In contrast, weaker hydrophobic interactions tend to accelerate and facilitate this process, allowing the steroids to “slide” into the binding pocket until the lactone ring engages with the water molecule stabilized by magnesium.

A comparison between α-L-rhamnosyl-ouabagenin (ouabain) and α-L-rhamnosyl-digitoxigenin (a digitoxigenin conjugated to rhamnose), in which the primary difference lies in the number of hydroxyl groups on the steroidal core, illustrates this effect. Although both molecules exhibit slow dissociation rates, the latter displays a faster association rate (Azalim-Neto et al. 2024) and, consequently, a higher affinity.

The number of hydroxyl groups in the steroidal core can be strongly influenced by pH, as demonstrated by Cornelius et al. (2013). In this study, ouabain (bearing five hydroxyl groups) showed a marked decrease in inhibitory potency (K_I_) for shark NKA pre-incubated for 1 h at 23 °C in Mg-Pi medium (which stabilizes the E2P conformation), with values shifting from 0.086 µM at pH 6.5 to 1.18 µM at pH 8.5 (a ~ 13-fold decrease). In contrast, digitoxin (with only the hydroxyl group at C14) exhibited a smaller change, from 0.183 µM at pH 6.5 to 0.404 µM at pH 8.5 (a ~ 2-fold decrease).

This effect became even more pronounced when comparing their aglycone counterparts: ouabagenin shifted from 0.404 µM at pH 6.5 to 32.1 µM at pH 8.5 (a ~ 80-fold decrease), whereas digitoxigenin changed from 0.132 µM at pH 6.5 to 0.373 µM at pH 8.5 (a ~ 3-fold decrease). These results may suggest that, although CTS with more hydrophilic steroidal cores are highly sensitive to pH, the glycosidic moiety tends to buffer or protect the inhibitory constant of these molecules.

The hydroxyl group at the C14 position on the α-face of the steroidal core, in addition to being a defining feature of CTS, forms a conserved interaction with the T797 (on crystallography obtained from NKA of the pig *Sus scrofa domesticus*, or T806 on crystallography obtained from NKA of the spiny dogfish *Squalus acanthias*) residue of the protein (Laursen et al. 2013; Kanai et al. 2020). Cyclization between C14 and C15, observed in certain bufadienolides such as resibufogenin and marinobufagenin, tends to markedly reduce affinity and inhibitory potency due to reduce on association and an increase in the dissociation rate. However, the introduction of an acetyl group at C16, even in the presence of cyclization can increase the affinity as seen in cinobufagin (Azalim-Neto et al., 2020, 2024).

## Glycosylation

Some CTS have a conjugated group, typically a glycosyl moiety, which is oriented towards the extracellular entrance of the binding site. These glycosyl groups do not engage in conserved hydrophilic interactions with the pocket due to their high flexibility and consequently low resolution on crystallography (Laursen et al. 2013, 2015).

Glycosidic substitutions at the C3 position represent one of the major natural modifications responsible for enhancing the affinity and inhibitory potency of CTS. For instance, it is well established that ouabain exhibits higher affinity than its aglycone counterpart, ouabagenin. Thus, glycosidic moieties constitute a major source of favorable entropy in these compounds. Therefore, glycosylation in CTS must be characterized with particular care, as it introduces substantial molecular complexity. This complexity arises both from its intrinsic energetic contribution, given the high conformational entropy (Fig. 9) associated with the large number of accessible rotamers (scaling approximately as 3ⁿ, where n is the number of dihedral angles), and from its role in protein interaction. Notably, this moiety enhances affinity not primarily through direct interactions, but by favorably modulating the dissociation dynamics.

Cornelius et al. (2013) demonstrated that even the addition of four digitoxose units does not drastically alter binding affinity compared to the aglycone counterpart. Despite this exceptionally high flexibility (potentially reaching up to 19,683 theoretical rotamers), it is not sufficient to significantly reduce the affinity of this CTS. This highlights how the NKA binding site accommodates, and even favors, the conformational entropy associated with C3 glycosylation. Such effects are often underestimated in molecular docking models, as the substantial conformational entropy suggests that the affinity and inhibitory potency of glycosylated steroids are not determined solely by the ground state observed in crystallographic structures.

Beyond the ground state, several dynamic phenomena associated with the glycosidic moiety may contribute to increased affinity in CTS. In addition to potentially facilitating the correct orientation of the steroidal scaffold (Laursen et al. 2013), other mechanisms may also be involved, such as avidity and protection against solvation. Avidity refers to the enhanced binding affinity arising from multiple, simultaneous interactions between a multivalent ligand and its receptor. For molecules bearing large conjugated groups, avidity provides a key mechanism for slow dissociation kinetics: even if the primary pharmacophore transiently detaches from its binding site, secondary contacts from the conjugated moiety maintain proximity, allowing rapid re-engagement before complete dissociation (Vauquelin and Charlton, 2010; 2013).

These properties also help to explain the enhanced α2 over α1 isoform selectivity observed for glycosylated derivatives compared with their aglycosylated counterparts, as identified by Katz and colleagues (Katz et al., 2014). The authors demonstrated that this selectivity increases when the interaction with residues of the β-subunit is optimized, leading to a reduced dissociation rate constant, even when no direct ground-state interaction is detected (Katz et al., 2015). This is consistent with crystallographic data showing that such direct interactions are not visible due to the high flexibility of the glycosylated moiety (Laursen et al. 2015). Because avidity is a dynamic phenomenon that contributes to the kinetic selectivity of slow-binding molecules (Copeland 2021), these observations reinforce the mechanistic basis for the distinct kinetics of glycosylated cardenolides compared to nonglycosylated ones.

Solvation of the ligand–receptor interaction may be one of the primary factors initiating dissociation of the complex. If the transition state is highly solvent-accessible, hydrophilic interactions between the protein residue and the ligand can be destabilized by a higher-energy water molecule, thereby triggering the dissociation process (Schmidtke et al. 2011). The presence of the glycosidic moiety may contribute to limiting water access, thus hindering this interaction and consequently slowing the dissociation process.

## Lactone ring

In the E2P ground state, the interaction of the lactone ring within the binding site can involve hydrogen bond acceptance from a water molecule stabilized by magnesium (however, for cardenolides at ~ 5 angstroms of distance). The butenolide ring of cardenolides is characterized by a strongly polarized carbonyl group, which increases the negative electron density on the carbonyl oxygen and, consequently, enhances its ability to act as a hydrogen bond acceptor compared to the α-pyrone ring of bufadienolides. However, in the E2P conformation, substitution of the butenolide ring with an α-pyrone does not significantly affect the affinity (Cornelius et al. 2013; Laursen et al. 2015; Noël et al. 2018; Azalim et al. 2020; Azalim-Neto et al. 2024). Notably, it is only upon potassium binding to ion sites II that class-dependent differences become apparent, highlighting the well-known antagonistic effect of potassium on cardenolides.

### NKA Conformational Change and Class Effect On Characterization

If the dynamics and interaction energetics of a ligand within a deep binding site are not sufficiently complex on their own, the protein additionally exhibits dynamic structural transitions throughout its catalytic cycle. Since NKA undergoes multiple conformational changes to bind and transport ions, the concentration of these ions in the incubation medium can shift the conformational equilibrium of the protein, affecting both CTS affinity (Laursen et al. 2015; Azalim et al. 2020; Kanai et al. 2020) and kinetics (Azalim-Neto et al. 2024).

The catalytic cycle of NKA causes drastic changes to the E2P conformation binding site due to potassium association. Initially, when the ion-binding site is empty, a single potassium ion binds to site I (Ogawa et al. 2015), allowing the reorganization of ion-binding site II to accommodate a second potassium ion (Kanai et al. 2020). In this phosphorylated conformation, the presence of potassium induces a rearrangement of the αTH1-4 (Laursen et al. 2015; Kanai et al. 2020), which can affect CTS binding/inhibition.

**Fig. 11:**
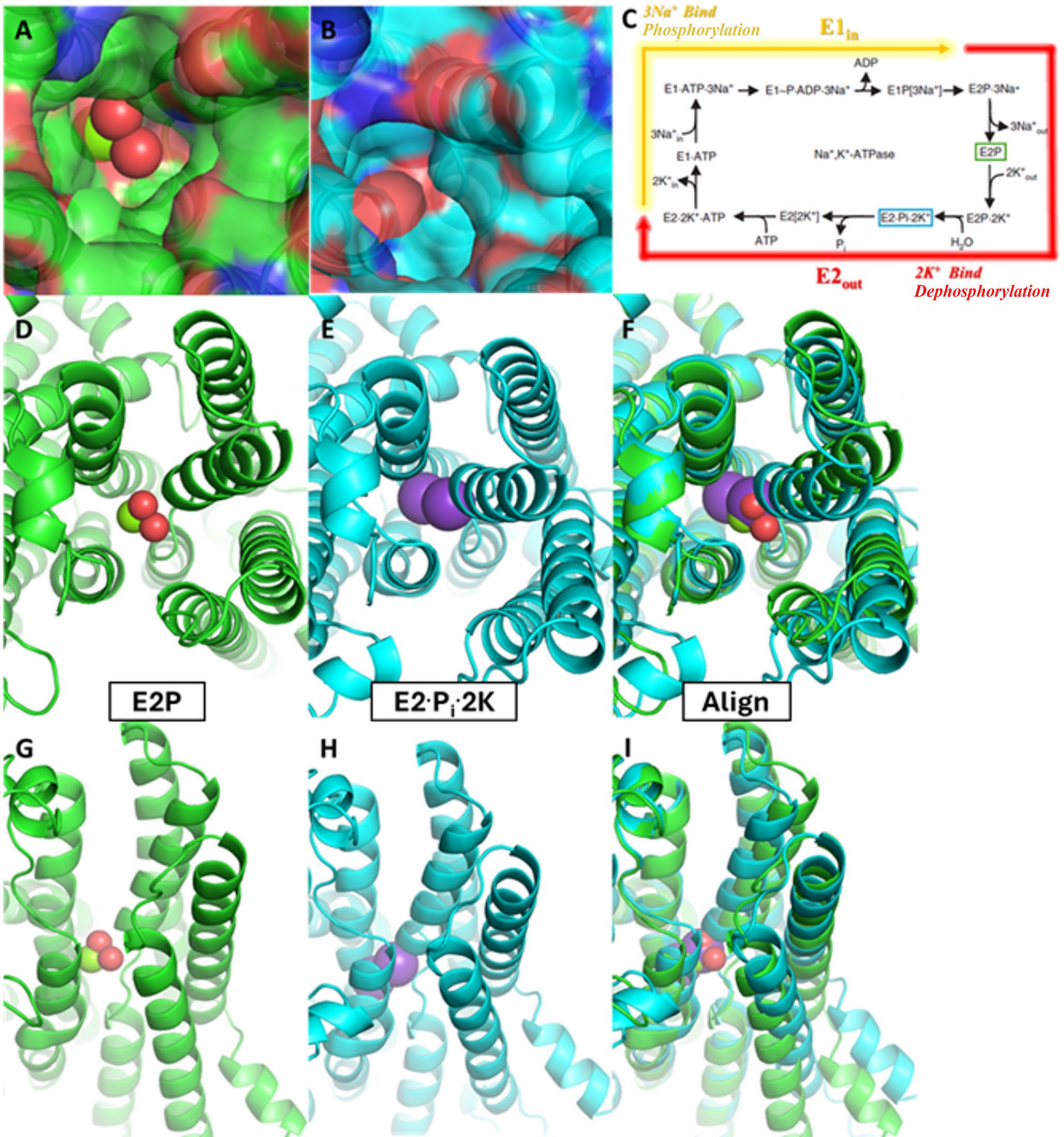
Conformational transition from E2P to E2–2 K^+^P_i_ (PDB ID: 7WYW and 2ZXE from *Squalus acanthias*, respectively). The extracellular cavity, viewed from the β-subunit, is represented by the solvent-accessible surface of the E2P conformation (**A**) and the E2P–2 K conformation (**B**). The Post–Albers catalytic cycle of Na^+^/K^+^-ATPase is shown in (**C**) (Post et al. 1972; Taniguchi and Post, 1975; source: adapted from Ogawa et al. 2015, https://creativecommons.org/licenses/by/4.0/). Cartoon representations of the transmembrane helices, viewed from the β-subunit, are shown for the E2P conformation (**D**), the E2P–2 K conformation (**E**), and the structural alignment of both states (**F**). A lateral view of the transmembrane helices is presented for the E2P conformation (**G**), the E2–2 K^+^P_i_ conformation (**H**), and the alignment of the two structures (**I**)

The cycle consists of two main conformations, based on the descriptions of ATPase transport by R. W. Albers (Albers 1967) and the specific descriptions of NKA ion transport by R. L. Post (Post et al. 1972), shown in Fig. 11. While the E1 conformation is characterized by the ion binding site being open to the intracellular space, allowing the binding of three sodium ions, and the phosphoryl transfer from ATP to the catalytic aspartate on the cytoplasmic region of the α subunit, it induces a conformational change to E2. The E2 conformation, in contrast, is characterized by the ion binding site opening to the extracellular space, allowing two potassium ions to bind and inducing a conformational change that leads to hydrolysis of phosphorylated aspartate (dephosphorylation). The association of CTS occurs with higher affinity when NKA is in the phosphorylated E2 state, the E2P conformation (Yatime et al. 2011).

As the E2P is the conformation with the binding site more accessible to the extracellular space, the association rate of all CTS will be higher on this conformation. When the protein goes from E2P to E2-2 K^+^P_i_, the cavity is less accessible, and the association rate of all CTS drops, reducing its affinity/inhibition (Azalim-Neto et al. 2024). Ouabain is the cardiotonic steroid most strongly affected by the transition from the E2P to the E2-2 K^+^P_i_ conformation. This conformational change destabilizes the αTH1-4 region, where most hydrogen bond interactions occur with the β-face of the steroidal core of ouabain, and its butanolide is dragged to αTH3-4.

**Fig. 12:**
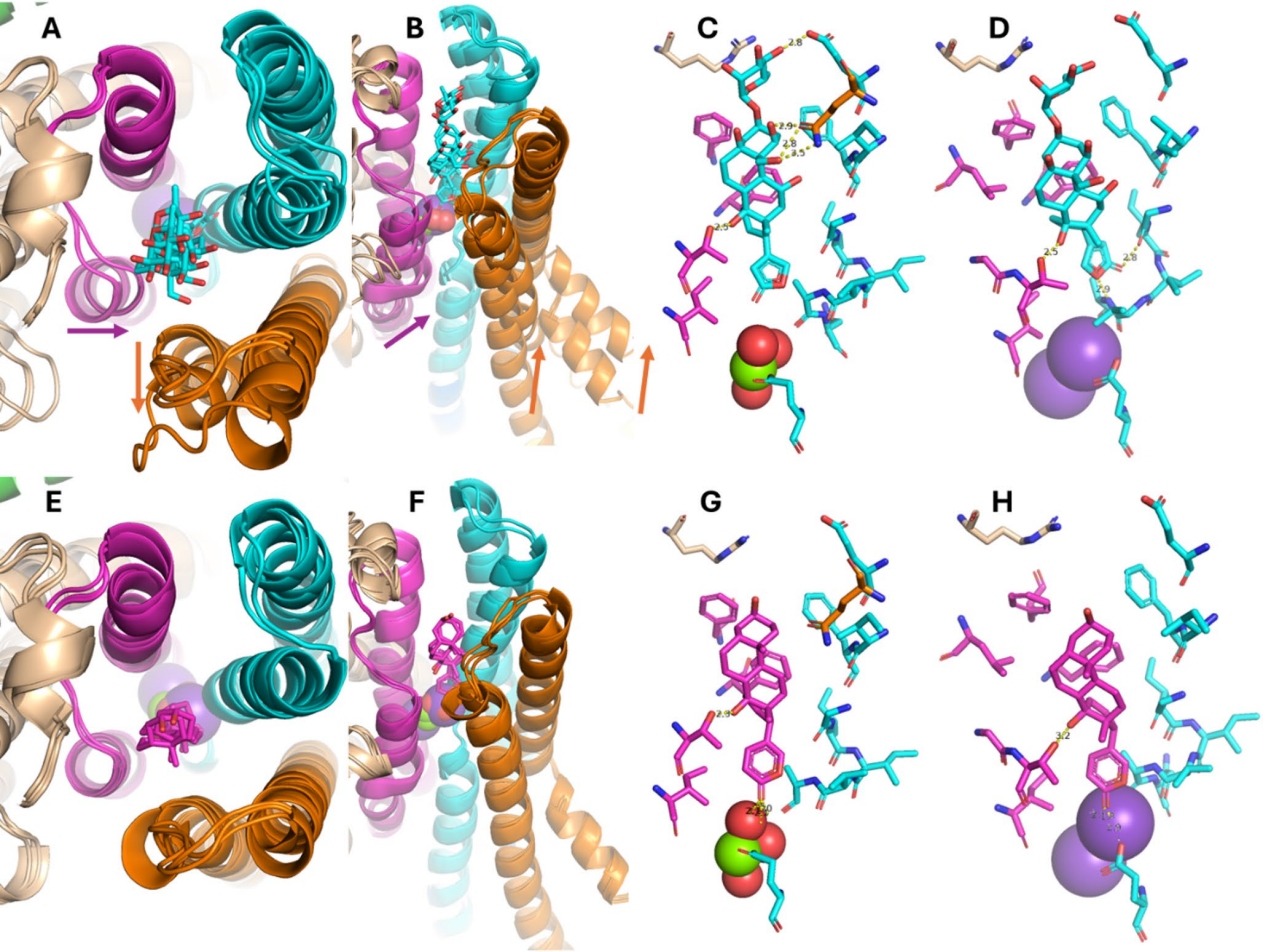
Conformational effects on CTS binding. In (**A**) (view from the β-subunit) and (**B**) (lateral view), alignment of Na^+^/K^+^-ATPase in the E2P conformation without ligand (7WYW from *Squalus acanthias*), E2P in the presence of ouabain (7WZ0 from *S. acanthias*), and the E2-2 K^+^P_i_ conformation in the presence of ouabain (3A3Y from *S. acanthias*) is shown. In (**C**) and (**D**), residues surrounding ouabain in the E2P and E2-2 K^+^P_i_ conformations, respectively, are presented. In (**E**) (view from the β-subunit) and (**F**) (lateral view), alignment of NKA in the E2P conformation (7WYW from *S. acanthias*) without ligand, E2P in the presence of bufalin (7DDL from *Sus scrofa*), and the E2P-2 K conformation in the presence of bufalin (4RES from *S. scrofa*) is shown. In (**G**) and (**H**), residues surrounding bufalin in the E2P and E2P-2 K conformations, respectively, are depicted. Colored arrows indicate the movement associated with the conformational changes of the respective transmembrane helices

In contrast, when considering a more hydrophobic steroidal core, the effect of potassium is reduced, as there is less dependence on hydrogen bond formation beyond the conserved C14–T797 (or T806 on *Squalus acanthias*) interaction. The α-pyrone ring, unlike the butenolide, becomes more effective in interacting with potassium or other closed residue; with more prevalence of conformation with potassium bounded, the dissociation rate of bufalin, previously similar to that of digitoxigenin, becomes slower (Azalim-Neto et al., 2024), maintaining its affinity, as both the association and dissociation rates are reduced (in contrast to ouabain, for which only the association rate is reduced) (Fig. 12F). However, at this moment, there is no E2-2 K^+^Pi conformation crystallography with bound bufalin. Consequently, it is not possible to conclude how the bufalin stays bound under this conformational change, as we have with ouabain. At the same time, it has no crystallography ouabain bound to E2P-2 K conformation.

Based on this framework, it is essential, not only for proper characterization but also for comparing results reported by different authors, that all incubation conditions be rigorously observed before concluding affinity or potency. In some cases, a pair of molecules described by one study may appear to have the same affinity, whereas another study reports different affinities for the same pair; both observations may, in fact, be correct. When the underlying mechanisms are dissected, both will converge with the same energetic and kinetic interpretation.

As discussed in the context of kinetic and thermodynamic characterization, molecules can reach the same affinity through distinct pathways. In this way, incubation conditions as time predominantly affect slow-binding CTS; pH has a greater impact on CTS with more hydrophilic steroidal cores; temperature more strongly influences entropy-driven CTS; and ion concentration, particularly potassium, has a more pronounced effect on cardenolides. Therefore, for CTS-NKA interaction, all these incubation parameters must be carefully considered when interpreting binding affinity and inhibitory potency.

## Model of CTS-NKA Interaction on Characterization

Thus far, we have considered that a one-phase model adequately describes the CTS–NKA interaction. However, the induced fit hypothesis remains the most widely used explanation for mechanisms involving the reduction of dissociation rates. In this model, when the ligand encounters the receptor, the latter undergoes a conformational change that promotes greater steric and electronic complementarity. Initially, the drug–receptor binding process is characterized by an initial equilibrium constant (*K*), derived from an initial association (*k*₊₁) and dissociation (*k*₋₁) rate constants. Subsequently, the ligand induces a conformational change in the receptor, generating a final equilibrium constant (*K⁎*), also characterized by final association (*k*₊₂) and dissociation (*k*₋₂) rate constants, strengthening the interaction by decreasing the dissociation rate (Copeland 2016; 2021; Vauquelin 2016).

The binding model of CTS on NKA, however, remains under debate, even in the presence of crystallographic data. Some authors argue that the lack of significant conformational changes in NKA, when comparing the binding of specific CTS to the unbound form within the same experiment, suggests the absence of an induced fit mechanism (Kanai et al. 2020). On the other hand, other studies have shown that conformational changes are present by comparing different crystallographic datasets of CTS–NKA interactions (Laursen et al. 2015). The main problem with crystallographic data (and with several other physical methods, such as calorimetry and surface plasmon resonance – SPR -, when applied to NKA) lies in the strong membrane anchoring of the protein and its large atomic structure, which contributes to the low resolution of the crystal structure. This compromises the ability to detect subtle conformational changes, mainly because when the changes are small, they may be completely masked by the resolution limit.

If the CTS–NKA interaction does not follow an induced fit model, the alternative is conformational selection. CTS can bind to different conformations of NKA with distinct affinities (Azalim et al. 2020) and kinetics (Azalim-Neto et al. 2024), with the highest affinity being observed for the E2P conformation. While it is still unclear whether CTS itself can induce a conformational change in NKA upon binding, it is well established that ions can cause such changes, thereby continuing the enzymatic cycle (Laursen et al. 2015; Kanai et al. 2020). This shows that conformational changes initiated via the binding site are possible, though perhaps not through the traditional interaction and energy promoted by CTS.

However, it is crucial to look beyond these models. As Miller et al. (2012) show when examining the molecular characteristics of ligands associated with slow *k*_off_ values, according to Lipinski’s Rule of Five (Lipinski et al. 1997), compounds with low molecular weight (< 300 Da) rarely exhibit slow dissociation rates unless they possess strong charge–charge interactions; on the other hand, molecules with molecular weights above 500 Da, high lipophilicity (clogP > 5), and a large number of rotatable bonds are more likely to exhibit slow kinetics, what is very compatible with the nature of CTS.

At the same time, it is also important to consider how the accessibility of the binding pocket influences association rates. Kinetic rates tend to be slower for molecules that must access a pocket via a loop displacement or a narrow channel, as opposed to those binding to an open, superficial, or helix-based site (Pan et al. 2013), and the deep binding site is the mark of NKA. However, some slower entropically interaction rates can occur if the ligand must induce a structural change, such as ordering a loop into a helix. Binding to flexible loop regions increases the likelihood of dissociation due to their dynamic nature. In contrast, binding directly to a rigid helix results in interaction rates that depend primarily on the interaction strength (Amaral et al. 2017), that is different from CTS-NKA interaction that is surrounded by helix.

**Fig. 13:**
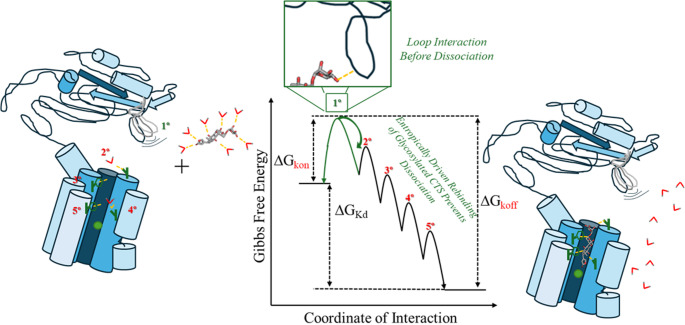
The image illustrates the energy and the rate of complex interaction between the cardiotonic steroid interaction and the Na^+^/K^+^-ATPase. The equilibrium of CTS is characterized mainly by the narrow and deep binding site, which finally reaches the ground state. The slow equilibrium of the glycosylated molecule can be due to the protection of water solvation and the possible loop interaction on β subunit, which may culminate in the rebinding of glycosylated molecules before it fully dissociates

In this way, multiple individual factors may contribute to the slowed equilibrium observed for specific CTS, especially glycosylated ones, as discussed previously. These factors include: the deep and narrow pathway leading to the binding site; the difficulty of solvent molecules (e.g., water) to solvate the ligand to induce the dissociation; and the presence of the β-subunit loop that partially covers the exit path from the binding site. This last factor, for example, may interact with glycosylated CTS and allow the molecule to transiently rebind before entirely exiting the binding site (Fig. 13), something that static structural techniques like crystallography may cannot capture. All these contributions cumulatively result in a slowdown of the equilibrium kinetics for some CTS, producing an effect like that seen in induced fit mechanisms in other drug–receptor systems.

## Key Considerations for Parameter Determinations on K^+^-pNPPase Inhibition Assays

Although the kinetic and thermodynamic characterization of CTS using K^+^-dependent phosphatase activity (K^+^-pNPPase) offers advantages, since the product pNP exhibits intrinsic absorbance and can be readily quantified without the need to stop the reaction or employ a coupled assay system, several limitations and challenges must be considered to properly interpret the properties of these inhibitors.

This K^+^-pNPPase activity typically represents ~ 20% of the ATPase activity, although it may vary depending on the ionic conditions employed (as it is stimulated by both potassium and magnesium). This is largely due to the lower affinity of pNPP (in the millimolar range) compared to ATP (which lies in the micromolar range). For preparations with high enzymatic activity, such as those obtained on a large scale through differential centrifugation and detergent treatment, this reduction in activity can be compensated by the high sensitivity of the pNP signal at 405 nm.

For cellular or more crude preparations, this signal sensitivity may not compensate for the low enzymatic activity, thereby substantially increasing imprecision when determining inhibition based on differences in total activity, which will already be low. This imprecision, arising from low-activity conditions, becomes a critical issue in the characterization of a molecule. This low activity, however, can also be compensated for by increasing the incubation time, since the lower substrate turnover compared to ATP allows the reaction to remain linear for a much longer period, being limited primarily by protein stability.

Unlike endpoint assays, monitoring activity progression allows one to verify whether the reaction remains linear over time, thereby avoiding misleading conclusions regarding the inhibitory behavior of the tested molecule. However, incubation time must primarily account for the interaction kinetics of the molecule, as this factor tends to influence equilibrium values more significantly than deviations from linearity (e.g., those caused by substrate hydrolysis exceeding ~ 5% of the total substrate). For fast-binding molecules, a higher data acquisition frequency is essential.

In principle, inhibition progress can be evaluated using only two concentrations (or even a single concentration, if it is close to 50% inhibition at equilibrium; however, this approach may mask cooperative behavior, if present). Moreover, selecting only two concentrations requires prior knowledge of the compound’s activity, which is rarely available when working with newly synthesized molecules. Therefore, using six concentrations in a 10-fold serial dilution is a suitable strategy to identify the active range of the compound in an initial experiment (aimed at characterization rather than screening).

Unlike simulated data, global fitting of experimental datasets with this method may involve considerable imprecision, mainly due to two factors: (1) low activity at early time points; and (2) conditions in which the tested compound exhibits little to no activity. In the latter case, imprecision is further amplified when working with percentage inhibition.

**Fig. 14:**
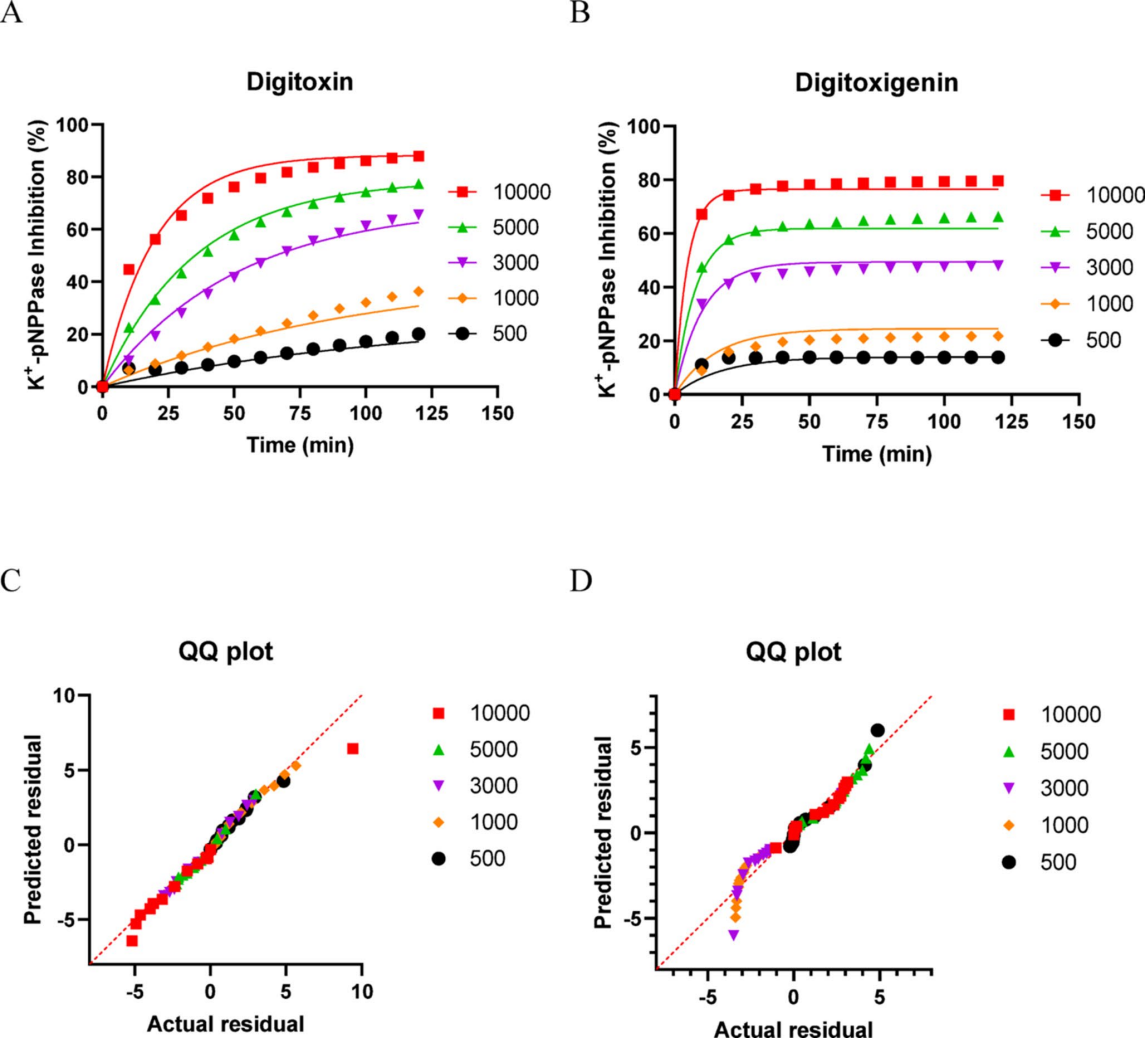
Quality of the fit (using the equation of *two or more concentration of htnm* present on GraphPad Prism) obtained by least-squares minimization of experiments performed with the following incubation conditions (in mM): 50 of KCl, 3 of MgCl_2_, 10 of pNPP, and 20 of Maleate-Tris pH 7.4 at 37 °C. A and B, inhibition progression curve of digitoxin (*k*_off_ = 0.006 min^− 1^, *k*_on_ = 4500 M^− 1^.min^− 1^, *K*_d_ = 1.3 × 10^− 6^ M) and digitoxigenin (*k*_off_ = 0.05 min^− 1^, *k*_on_ = 16098 M^− 1^.min^− 1^, *K*_d_ = 3 × 10^− 6^ M). The concentrations are presented in nM. C and D, QQ plot of the residuals of the respective CTS, indicating that lower concentrations and/or early time points were primarily responsible for deviations from normality

As illustrated in Fig. 14, although the residuals generally display normality, some points begin to deviate from this pattern, suggesting an inadequate fit of the equation for those experimental points. However, it is possible to identify from the fit that the primary sources of imprecision arise from the early time points and from compound concentrations that are weakly active (~ 15% inhibition) or inactive.

This situation is exacerbated when characterizing kinetics at lower temperatures (~ 20 °C), as overall activity decreases, bringing total activity closer to basal levels and increasing imprecision at early time points. In such cases, if more than one concentration fails to fit properly (i.e., does not capture the inflection), the experiment should be repeated.

Conversely, the opposite occurs at higher temperatures (~ 45 °C), where activity is increased and may rapidly approach saturation. For some molecules, equilibrium may be reached very quickly, within less than 5 min, requiring automated pipetting and data acquisition at short time intervals (on the order of seconds). If the inflection is not adequately captured due to insufficient sampling frequency, imprecision increases, and the kinetic parameters cannot be reliably determined.

Finally, all kinetic and thermodynamic parameters obtained should be treated carefully before performing the SAR description or the Quantitative SAR (QSAR) analysis. Although many population parameters follow a normal distribution, in some biological fields, where we describe tissues, cells, or protein functions such as expression, affinity, or enzyme velocity, normal distribution is less common, and skewed distributions tend to arise (Motulsky, 2017).

In such cases, reporting values such as *K*_d_, *k*_on_, and *k*_off_ as means ± standard deviation (SD) is a mistake, since these values follow a log-normal distribution (Hancock et al. 1988), different from the thermodynamic parameters. The mean does not accurately reflect the central tendency, and the standard deviation cannot appropriately represent dispersion due to the asymmetry of the data. Furthermore, parametric methods (e.g., *t*-test, ANOVA, Pearson correlation) cannot be applied, as they rely strictly on the assumption of normality.

Descriptive statistics for log-normally distributed parameters should be reported as the geometric mean for central tendency, as it uses the log-transformation of the data. The dispersion in this case can only be expressed with the SD of its log, as only its log follows a normal distribution (for example: *K*_d_ = 10^− 9±0.5^M). It is possible to report the confidence intervals (however, it is not the dispersion of the data, which is a measure of precision of the mean), to preserve the original scale of the data. Reporting both log-transformed mean ± SD values is less intuitive for inclusion in tables. Alternatively, a nonparametric approach can be used, reporting the median (from the ordered dataset, as it is literally the concept of central tendency) as central tendency and the interquartile range as dispersion (Mishra et al. 2019), although this is less commonly done. For inferential statistics, parametric tests may still be used if the parameters are log-transformed (p*K*_d_, p*k*_on_ and p*k*_off_). Comparisons between groups (e.g., *t*-test and ANOVA) or correlation analyses (e.g., Pearson) can then be appropriately conducted on the transformed data.

### Limitations and Difficulties in CTS-NKA Kinetics and Thermodynamics Characterization

Although the use of Eyring’s linear equation is an accessible method to infer thermodynamics for enzyme inhibitors, it has significant limitations. Both enthalpy and entropy are estimated in the same experiment via linear regression (Khrapunov 2018), which may introduce bias and falsely characterize a substance as being entropically driven compared to more direct methods like calorimetry (Winquist et al. 2013).

The alternative for kinetic and thermodynamic characterization is the direct methods like SPR for kinetics and Isothermal Titration Calorimetry (ITC) for thermodynamics. Although the primary method for direct kinetic characterization is SPR, a label-free technique in which the target protein is immobilized on the surface of a sensor, and the ligand is added to a flow system (He et al. 2006; Karlsson et al. 2006; Rich and Myszka 2010), several challenges arise in the case of membrane proteins. These include difficulties in expression, purification, membrane reconstitution, and, especially, in immobilizing a high density of membrane proteins required for the use of low ligand concentrations. Such challenges explain the limited number of SPR studies involving membrane proteins (Stenlund et al. 2003; Congreve et al. 2011; Patching 2014). Since NKA is an integral membrane protein, the use of SPR becomes especially limited, and the few available studies report low affinity of the well-known high-affinity CTS (Alfonso et al. 2013; Liu et al. 2016).

The same problem can appear for direct determination of binding enthalpy with ITC, which allows for reliable measurement of enthalpy and, from it, the derivation of Gibbs free energy and entropy. However, in some cases, particularly in interactions between membrane proteins and compounds dissolved in solvents with a high heat of dilution, this measurement becomes unfeasible (Borea et al. 2000; Ladbury 2004).

When comparing calorimetric data for CTS, however, some studies do not show entropic dominance for substances previously described as “slow binders” in the literature (Klimanova et al. 2015; Tverskoi et al. 2021). Conversely, other studies have reported entropic dominance for ouabain, but only at much lower affinity (1.2 mM at 25 °C) (Adzhubei et al. 2022), or even the opposite scenario, where fast kinetics were entropically driven (Cornelius et al. 2013).

These conflicting findings arise from numerous experimental differences, such as the use of different animal species as source material, distinct ionic conditions during incubation, and particularly the intervals between injections. This last parameter is a fundamental variable when determining the thermodynamic profile of slow-binding CTS (Grell et al. 1997, 2001; Stolz et al. 2003), and it has also been used to characterize association and dissociation rate constants in calorimetric studies (Dumas et al. 2016).

Given the limited number of calorimetric studies and the significant variability in their results, as well as the methodological challenges of validating approaches such as Van’t Hoff and Eyring, it is not easy to draw definitive conclusions regarding the thermodynamic signatures of slow- versus fast-binding CTS. It is possible, for instance, that a molecule may be enthalpically driven in one enzyme conformation, but entropically driven in another.

Moreover, the fact that glycosylated CTS exhibit slow association and dissociation rates and appear to be entropically driven is consistent with their flexibility, deep binding site, and possibly the ability to resist dissociation induced by water molecules, leading to a highly unfavorable dissociation transition state, a phenomenon also observed in other drug–receptor systems (Schmidtke et al. 2011).

### Future Perspectives for CTS Efficacy

Regarding the CTS–NKA interaction and its efficacy, a direct influence of kinetics was first suggested by Lüllmann et al. (1984). According to the authors, ouabain exhibits an inhibitory potency on NKA nearly 100 times greater than that of 3-α-methyl-digitoxigenin-glycoside, yet both compounds show a very similar maximum inhibition (less than a 10% difference). However, when comparing the inotropic effect, ouabain is 10 times more potent than methyl-digitoxigenin-glycoside, but its maximum inotropic effect reaches only 65%, compared to 110% for methyl-digitoxigenin-glycoside. They found that while ouabain exhibited slow binding kinetics, methyl-digitoxigenin-glycoside showed much faster binding kinetics. Based on these findings, the authors concluded that the positive inotropic effect induced by CTS could be mediated by the frequency of interaction with NKA rather than by their inhibitory potency on the enzyme’s activity.

As the signaling effect resulting from the CTS–NKA interaction has been investigated in various contexts (as stated here in the Introduction section), one emerging point of interest is the investigation of functional selectivity, the presence of distinct effects produced by different CTS (Banerjee et al., 2021; Xu et al., 2021). Amaral et al. (2018) demonstrated that telocinobufagin and marinobufagenin produced distinct cellular effects in LLC-PK1 cells: while both compounds increased ERK1/2 phosphorylation, telocinobufagin induced cell proliferation, whereas marinobufagenin increased the sub-G0 cell cycle phase, leading to enhanced apoptosis. We have shown that these two CTS exhibit different kinetics – though, identified in a semi-quantitative manner using t_1/2_ of one concentration at equilibrium close to the *K*_i_ value (t_1/2_ min = 51.3 and 4.7 for telocinobufagin and marinobufagin, respectively) – on K^+^-pNPPase inhibition (Azalim et al. 2020), and this kinetic behavior could be crucial to better characterize the possible functional selectivity of this interaction. However, many of these studies still assume the induced fit model to explain biased signaling, even though this remains debated in crystallographic data.

Indeed, biased signaling is defined as the ligand-dependent activation of certain pathways over others, leading to a functionally selective response, as seen with many drugs interacting with G protein-coupled receptors (Kolb et al. 2022). Therefore, for its “functional selectivity” to be characterized for CTS, the influence of the rate of interaction with the NKA could be assessed to investigate the impact of the rate on the most important second messenger signal induced by CTS, that is, the intracellular calcium. CTS can also induce slow calcium oscillations rather than just a sustained increase in intracellular calcium concentration, as shown by Anita Aperia’s group (Fontana et al., 2013). Both amplitude and frequency of intracellular calcium can modulate the activity of cell factors, such as NF-κB (Ruffinatti et al. 2011; Smedler and Uhlén 2014).

Many works have focused on describing the effect of ouabain on the modulation of NF-kB, as the drug can increase the activation of this factor in rat cerebellar cell culture (de Sá Lima et al. 2013), but can be reduced in inflammatory models of peritonitis in mice (Leite et al. 2015). However, the glycosylated CTS has gained more focus than its aglycosylated pair. As we discussed, this chemical modification can bring large differences to the kinetic behavior of CTS, sometimes even without affecting its affinity. It will be interesting to identify if changing *k*_on_ or *k*_off_ can affect the selectivity and the modulation of effect, consequently, understand how the chemical modifications on rate, more than affinity, can help to better describe the myriad effects produced by CTS on cells.

### Concluding Remarks

Because the CTS–NKA interaction involves a deep binding site undergoing continuous conformational changes, dynamically altering its composition and accommodating structurally complex, entropy-driven ligands, determining both the energetic and kinetic components is essential for proper characterization of this complex.

Characterization of CTS using the K^+^-pNPPase inhibition assay can be highly informative due to its scalability, provided that its limitations are carefully considered, as it represents only a fraction of the overall ATPase activity. And as a statistical consideration, before performing data analysis (descriptive or inferential), it is essential to consider that pharmacological and biochemical parameters frequently follow a log-normal distribution.

As models of CTS–NKA interactions remain under debate, data fitting should initially prioritize the one-step model, while accounting for the individual contributions of conjugated moieties and the influence of distinct protein conformations, rather than prematurely adopting more complex fitting models.

A deeper understanding of the kinetic and thermodynamic aspects of CTS–NKA interactions is expected to provide a more comprehensive basis for interpreting the efficacy and selectivity of these molecules in the literature.

## Data Availability

No datasets were generated or analysed during the current study.

## References

[CR1] Adzhubei AA, Tolstova AP, Strelkova MA, Mitkevich VA, Petrushanko IY, Makarov AA (2022) Interaction Interface of Aβ42 with Human Na,K-ATPase Studied by MD and ITC and Inhibitor Screening by MD. Biomedicines 10. 10.3390/biomedicines10071663 10.3390/biomedicines10071663 PMC9313104 35884966

[CR2] Albers RW (1967) Biochemical aspects of active transport. Annu Rev Biochem 36:727–756 Available at: www.annualreviews.org 18257736 10.1146/annurev.bi.36.070167.003455

[CR3] Alfonso A, Pazos MJ, Fernández-Araujo A, Tobio A, Alfonso C, Vieytes MR et al (2013) Surface plasmon resonance biosensor method for palytoxin detection based on Na+,K+-ATPase affinity. Toxins (Basel) 6:96–107. 10.3390/toxins6010096 24379088 10.3390/toxins6010096 PMC3920252

[CR5] Amaral M, Kokh DB, Bomke J, Wegener A, Buchstaller HP, Eggenweiler HM et al (2017) Protein conformational flexibility modulates kinetics and thermodynamics of drug binding. Nat Commun 8. 10.1038/s41467-017-02258-w 10.1038/s41467-017-02258-w PMC5741624 29273709

[CR4] Amaral LS, Ferreira JM, Predes D, Abreu JG, Noël F, Quintas LEM (2018) Telocinobufagin and marinobufagin produce different effects in LLC-PK1 cells: A case of functional selectivity of bufadienolides. Int J Mol Sci 19. 10.3390/ijms19092769 10.3390/ijms19092769 PMC6163863 30223494

[CR6] Arrhenius S (1887) Über die Dissociation der im Wasser gelösten Stoffe. Z für Phys Chemie 1(1):631–648

[CR7] Atkins P, De Paula J (2017) Teoria do estado de transição. in Físico-Química. LTC), (Rio de Janeiro, pp 763–774

[CR8] Azalim P, do Monte FM, Rendeiro MM, Liu X, O’Doherty GA, Fontes CF et al (2020) Conformational states of the pig kidney Na+/K+-ATPase differently affect bufadienolides and cardenolides: A directed structure-activity and structure-kinetics study. Biochem Pharmacol 171:113679. 10.1016/j.bcp.2019.113679 31669257 10.1016/j.bcp.2019.113679

[CR9] Azalim-Neto P, Noël F, Silva SC, Villar JAFP, Barbosa L, O’Doherty GA et al (2024) Simplified method for kinetic and thermodynamic screening of cardiotonic steroids through the K^+^-dependent phosphatase activity of Na+/K+-ATPase with chromogenic pNPP substrate. Mol Pharmacol 106:225–239. 10.1124/molpharm.124.000934 39187390 10.1124/molpharm.124.000934 PMC11493336

[CR10] Babula P, Masarik M, Adam V, Provaznik I, Kizek R (2013) From Na + /K +-ATPase and cardiac glycosides to cytotoxicity and cancer treatment. Anticancer Agents Med Chem. 13(7):1069–1087 10.2174/18715206113139990304 23537048

[CR110] Banerjee M, Li Z, Gao Y, Lai F, Huang M, Zhang Z, Cai L, Sanabria J, Gao T, Xie Z, Pierre SV (2021) Inverse agonism at the Na/K-ATPase receptor reverses EMT in prostate cancer cells. Prostate 81:667–682. 10.1002/pros.24144 10.1002/pros.24144 PMC10071553 33956349

[CR11] Borea PA, Dalpiaz A, Varani K, Gilli P, Gilli G (2000) Can thermodynamic measurements of receptor binding yield information on drug affinity and efficacy? Biochem Pharmacol 60:1549–1556. 10.1016/S0006-2952(00)00368-3 11077036 10.1016/s0006-2952(00)00368-3

[CR12] Clark AJ (1933) The mode of action of drugs on cells. Edward Arnold & Co, London

[CR13] Congreve M, Rich RL, Myszka DG, Figaroa F, Siegal G, Marshall FH (2011) Fragment screening of stabilized G-protein-coupled receptors using biophysical methods. in Methods in Enzymology. Academic Press Inc., pp 115–136. doi: 10.1016/B978-0-12-381274-2.00005-4 10.1016/B978-0-12-381274-2.00005-4 21371589

[CR14] Copeland RA (2016) The drug-target residence time model: A 10-year retrospective. Nat Rev Drug Discov 15:87–95. 10.1038/nrd.2015.18 26678621 10.1038/nrd.2015.18

[CR15] Copeland RA (2021) Evolution of the drug-target residence time model. Expert Opin Drug Discov 16:1441–1451. 10.1080/17460441.2021.1948997 34210223 10.1080/17460441.2021.1948997

[CR16] Cornelius F, Kanai R, Toyoshima C (2013) A structural view on the functional importance of the sugar moiety and steroid hydroxyls of cardiotonic steroids in binding to Na,K-ATPase. J Biol Chem 288:6602–6616. 10.1074/jbc.M112.442137 23341448 10.1074/jbc.M112.442137 PMC3585100

[CR500] de Oliveira GC, Rocha SC, da Silva Lopes MA, Paixão N, Alves SLG, Pessoa MTC, Noël F, Quintas LEM, Barbosa LA, Villar JAFP, Cortes VF (2021) Implications of Synthetic Modifications of the Cardiotonic Steroid Lactone Ring on Cytotoxicity. J Membr Biol. 254(5-6):487–497. 10.1007/s00232-021-00186-x. 10.1007/s00232-021-00186-x 34128090

[CR17] de Sá Lima L, Kawamoto EM, Munhoz CD, Kinoshita PF, Orellana AMM, Curi R et al (2013) Ouabain activates NFκB through an NMDA signaling pathway in cultured cerebellar cells. Neuropharmacology 73:327–336. 10.1016/j.neuropharm.2013.06.006 23774137 10.1016/j.neuropharm.2013.06.006

[CR18] Dumas P, Ennifar E, Da Veiga C, Bec G, Palau W, Di Primo C et al (2016) Extending ITC to Kinetics with kinITC. in Methods in Enzymology. Academic Press Inc.), pp 157–180. doi: 10.1016/bs.mie.2015.08.026 10.1016/bs.mie.2015.08.026 26794354

[CR19] El-Seedi HR, Khalifa SAM, Taher EA, Farag MA, Saeed A, Gamal M et al (2019) Cardenolides: Insights from chemical structure and pharmacological utility. Pharmacol Res 141:123–175. 10.1016/j.phrs.2018.12.015 30579976 10.1016/j.phrs.2018.12.015

[CR20] Evans MG, Polanyi M (1935) Some applications of the transition state method to the calculation of reaction velocities, especially in solution. Trans Faraday Soc 31:875–894. 10.1039/tf9353100875

[CR21] Eyring H (1935) The activated complex in chemical reactions. J Chem Phys 3:107–115. 10.1063/1.1749604

[CR121] Fontana JM, Burlaka I, Khodus G, Brismar H, Aperia A (2013) Calcium oscillations triggered by cardiotonic steroids. FEBS J 280:5450–5455. 10.1111/febs.12448 10.1111/febs.12448 23890276

[CR22] Gibbs JW (1876–1878). On the equilibrium of heterogeneous substances. Trans Conn Acad Arts Sci, 3, 108–248, 343–524

[CR23] Grell E, Mutz M, Marti E (1997) Membrane-bound Na+,K+-ATPase as the cardiac glycoside receptor. J Therm Anal 48:437–445. 10.1007/BF01979490

[CR24] Grell E, Schick E, Lewitzki E (2001) Membrane receptor calorimetry: cardiac glycoside interaction with Na,K-ATPase. Thermochim Acta 380:245–254

[CR25] Hancock AA, Bush EN, Stanisic D, Kyncl JJ, Lin CT (1988) Data normalization before statistical analysis: keeping the horse before the cart. Trends Pharmacol Sci 9:29–32. 10.1016/0165-6147(88)90239-8 3245075 10.1016/0165-6147(88)90239-8

[CR26] Harper EA, Black JW (2007) Histamine H 3-receptor agonists and imidazole-based H 3-receptor antagonists can be thermodynamically discriminated. Br J Pharmacol 151:504–517. 10.1038/sj.bjp.0707243 17401438 10.1038/sj.bjp.0707243 PMC2013973

[CR27] He X, Coombs D, Myszka DG, Goldstein B (2006) A theoretical and experimental study of competition between solution and surface receptors for ligand in a biacore flow cell. Bull Math Biol 68:1125–1150. 10.1007/s11538-006-9093-9 16804651 10.1007/s11538-006-9093-9

[CR28] Hill AV (1910) The possible effects of the aggregation of the molecules of haemoglobin on its dissociation curves. J Physiol 40:iv–vii. 10.1113/jphysiol.1910.sp001386

[CR29] Kanai R, Cornelius F, Ogawa H, Motoyama K, Vilsen B, Toyoshima C (2020) Binding of cardiotonic steroids to Na+,K+-ATPase in the E2P state. PNAS 118. 10.1073/pnas.2020438118/-/DCSupplemental 10.1073/pnas.2020438118 PMC7817145 33318128

[CR30] Karlsson R, Katsamba PS, Nordin H, Pol E, Myszka DG (2006) Analyzing a kinetic titration series using affinity biosensors. Anal Biochem 349:136–147. 10.1016/j.ab.2005.09.034 16337141 10.1016/j.ab.2005.09.034

[CR102] Katz A, Tal DM, Heller D, Haviv H, Rabah B, Barkana Y, Marcovich AL, Karlish SJ (2014) Digoxin derivatives with enhanced selectivity for the α2 isoform of Na,K-ATPase: effects on intraocular pressure in rabbits. J Biol Chem 289(30):21153–21162. 10.1074/jbc.M114.557629 10.1074/jbc.M114.557629 PMC4110318 24917667

[CR103] Katz A, Tal DM, Heller D, Habeck M, Ben Zeev E, Rabah B, Bar Kana Y, Marcovich AL, Karlish SJD (2015) Digoxin derivatives with selectivity for the α2β3 isoform of Na,K-ATPase potently reduce intraocular pressure. Proc. Natl. Acad. Sci. U.S.A. 112(44):13723–13728. 10.1073/pnas.1514569112 10.1073/pnas.1514569112 PMC4640782 26483500

[CR31] Khrapunov S (2018) The Enthalpy-entropy Compensation Phenomenon. Limitations for the Use of Some Basic Thermodynamic Equations. Curr Protein Pept Sci 19:1088–1091. 10.2174/1389203719666180521092615 29779476 10.2174/1389203719666180521092615 PMC6142176

[CR32] Klimanova EA, Petrushanko IY, Mitkevich VA, Anashkina AA, Orlov SN, Makarov AA et al (2015) Binding of ouabain and marinobufagenin leads to different structural changes in Na,K-ATPase and depends on the enzyme conformation. FEBS Lett 589:2668–2674. 10.1016/j.febslet.2015.08.011 26297827 10.1016/j.febslet.2015.08.011

[CR33] Kolb P, Kenakin T, Alexander SPH, Bermudez M, Bohn LM, Breinholt CS et al (2022) Community guidelines for GPCR ligand bias: IUPHAR review 32. Br J Pharmacol 179:3651–3674. 10.1111/bph.15811 35106752 10.1111/bph.15811 PMC7612872

[CR34] Kumavath R, Paul S, Pavithran H, Paul MK, Ghosh P, Barh D et al (2021) Emergence of cardiac glycosides as potential drugs: Current and future scope for cancer therapeutics. Biomolecules 11. 10.3390/biom11091275 10.3390/biom11091275 PMC8465509 34572488

[CR35] Ladbury JE (2004) Application of Isothermal Titration Calorimetry in the Biological Sciences: Things Are Heating Up! Bio Techniques 37:885–887 10.2144/04376TE01 15597533

[CR36] Lafont V, Armstrong AA, Ohtaka H, Kiso Y, Amzel M, L., and, Freire E (2007) Compensating enthalpic and entropic changes hinder binding affinity optimization. Chem Biol Drug Des 69:413–422. 10.1111/j.1747-0285.2007.00519.x 17581235 10.1111/j.1747-0285.2007.00519.x

[CR38] Laursen M, Yatime L, Nissen P, Fedosova NU (2013) Crystal structure of the high-affinity Na+,K+-ATPase- ouabain complex with Mg2 + bound in the cation binding site. PNAS 110:10958–10963. 10.1073/pnas.1222308110 23776223 10.1073/pnas.1222308110 PMC3704003

[CR37] Laursen M, Gregersen JL, Yatime L, Nissen P, Fedosova NU (2015) Structures and characterization of digoxin- And bufalin-bound Na+,K+-ATPase compared with the ouabain-bound complex. PNAS 112:1755–1760. 10.1073/pnas.1422997112 25624492 10.1073/pnas.1422997112 PMC4330780

[CR39] Leite JA, Alves AKDA, Galvão JGM, Teixeira MP, Cavalcante-Silva LHA, Scavone C et al (2015) Ouabain Modulates Zymosan-Induced Peritonitis in Mice. Mediators Inflamm 2015. 10.1155/2015/265798 10.1155/2015/265798 PMC4442290 26078492

[CR40] Lipinski CA, Dominy BW, Feeney PJ (1997) Drug delivery reviews Experimental and computational approaches to estimate solubility and permeability in drug discovery and development settings. Adv Drug Deliv Rev 23:3–25 10.1016/s0169-409x(00)00129-0 11259830

[CR41] Liu M, Feng LX, Sun P, Liu W, Wu WY, Jiang BH et al (2016) A novel bufalin derivative exhibited stronger apoptosis-inducing effect than bufalin in A549 lung cancer cells and lower acute toxicity in mice. PLoS ONE 11. 10.1371/journal.pone.0159789 10.1371/journal.pone.0159789 PMC4961401 27459387

[CR202] Lüllmann H, Peters T, Prillwitz H-H, and Ziegler A (1984) Cardiac glycosides with different effects in the heart, in Cardiac Glycoside Receptors and Positive Inotropy pp 93–101, Steinkopff, Heidelberg. 10.1007/978-3-642-72376-6_13 6331383

[CR42] Maehle A-H (2005) The quantification and differentiation of the drug receptor theory, c. 1910–1960. Ann Sci 62:479–500. 10.1080/00033790412331312666 16482711 10.1080/00033790412331312666

[CR44] Mijatovic T, Roland I, Van Quaquebeke E, Nilsson B, Mathieu A, Van Vynckt F et al (2007) The α1 subunit of the sodium pump could represent a novel target to combat non-small cell lung cancers. J Pathol 212:170–179. 10.1002/path.2172 17471453 10.1002/path.2172

[CR43] Mijatovic T, Dufrasne F, Kiss R (2012) Cardiotonic Steroids-Mediated Targeting of the Na + /K +-ATPase to Combat Chemoresistant Cancers. Curr Med Chem 19:627–646 22204337 10.2174/092986712798992075

[CR45] Miller DC, Lunn G, Jones P, Sabnis Y, Davies NL, Driscoll P (2012) Investigation of the effect of molecular properties on the binding kinetics of a ligand to its biological target. Medchemcomm 3:449–452. 10.1039/c2md00270a

[CR46] Mishra P, Pandey C, Singh U, Gupta A, Sahu C, Keshri A (2019) Descriptive statistics and normality tests for statistical data. Ann Card Anaesth 22:67. 10.4103/aca.ACA_157_18 30648682 10.4103/aca.ACA_157_18 PMC6350423

[CR105] Motulsky H (2017) Intuitive Biostatistics: A Nonmathematical Guide to Statistical Thinking, 4th ed., Oxford University Press, New York, p. 91.

[CR47] Noël F (2017) Ensaios de Cinética: Determinação do tempo de residência, in *Ensaios de Binding: Fundamentos teóricos, aspectos práticos e aplicações no desenvolvimento de fármacos*, (Rio de Janeiro: SBFTE), 46–51. Available at: http://francoisnoelfarmac.wixsite.com/fnoel

[CR48] Noël F, Azalim P, do Monte FM, Quintas LEM, Katz A, Karlish SJD (2018) Revisiting the binding kinetics and inhibitory potency of cardiac glycosides on Na +,K + -ATPase (α1β1): Methodological considerations. J Pharmacol Toxicol Methods 94:64–72. 10.1016/j.vascn.2018.09.001 30244071 10.1016/j.vascn.2018.09.001

[CR49] Ogawa H, Cornelius F, Hirata A, Toyoshima C (2015) Sequential substitution of K+ bound to Na+,K+-ATPase visualized by X-ray crystallography. Nat Commun 6. 10.1038/ncomms9004 10.1038/ncomms9004 PMC4918401 26258479

[CR501] Ogawa H, Shinoda T, Cornelius F, Toyoshima C (2009) Crystal structure of the sodium-potassium pump (Na+,K+-ATPase) with bound potassium and ouabain. Proc Natl Acad Sci U S A. 106(33):13742–7. 10.1073/pnas.0907054106. 10.1073/pnas.0907054106 PMC2728964 19666591

[CR50] Pan AC, Borhani DW, Dror RO, Shaw DE (2013) Molecular determinants of drug–receptor binding kinetics. Drug Discov Today 18:667–673. 10.1016/j.drudis.2013.02.007 23454741 10.1016/j.drudis.2013.02.007

[CR51] Patching SG (2014) Surface plasmon resonance spectroscopy for characterisation of membrane protein-ligand interactions and its potential for drug discovery. Biochim Biophys Acta Biomembr 1838:43–55. 10.1016/j.bbamem.2013.04.028 10.1016/j.bbamem.2013.04.028 23665295

[CR52] Pessôa MTC, Valadares JMM, Rocha SC, Silva SC, McDermott JP, Sánchez G et al (2020) 21-Benzylidene digoxin decreases proliferation by inhibiting the EGFR/ERK signaling pathway and induces apoptosis in HeLa cells. Steroids 155:108551. 10.1016/j.steroids.2019.108551 31812624 10.1016/j.steroids.2019.108551 PMC7028499

[CR53] Post RL, Hegyvary C, Kume S (1972) Activation by adenosine triphosphate in the phosphorylation kinetics of sodium and potassium ion transport adenosine triphosphatase. J Biol Chem 247:6530–6540. 10.1016/s0021-9258(19)44725-x 4263199

[CR54] Radnai L, Stremel RF, Sellers JR, Rumbaugh G, Miller CA (2019) A semi-high-throughput adaptation of the NADH-coupled ATPase assay for screening small molecule inhibitors. *Journal of Visualized Experiments* 2019. 10.3791/60017 10.3791/60017 PMC7041180 31475972

[CR55] Rich RL, Myszka DG (2010) Grading the commercial optical biosensor literature - Class of 2008: The Mighty Binders. J Mol Recognit 23:1–64. 10.1002/jmr.1004 20017116 10.1002/jmr.1004

[CR56] Rocha SC, Pessoa MTC, Neves LDR, Alves SLG, Silva LM, Santos HL et al (2014) 21-Benzylidene digoxin: a proapoptotic cardenolide of cancer cells that up-regulates Na,K-ATPase and epithelial tight junctions. PLoS ONE 9:e108776. 10.1371/journal.pone.0108776 25290152 10.1371/journal.pone.0108776 PMC4188576

[CR57] Ruffinatti FA, Lovisolo D, Distasi C, Ariano P, Erriquez J, Ferraro M (2011) Calcium signals: Analysis in time and frequency domains. J Neurosci Methods 199:310–320. 10.1016/j.jneumeth.2011.05.009 21658413 10.1016/j.jneumeth.2011.05.009

[CR58] Sakai H, Suzuki T, Maeda M, Takahashi Y, Horikawa N, Minamimura T et al (2004) Up-regulation of Na+,K+-ATPase α3-isoform and down-regulation of the α1-isoform in human colorectal cancer. FEBS Lett 563:151–154. 10.1016/S0014-5793(04)00292-3 15063740 10.1016/S0014-5793(04)00292-3

[CR59] Schmidtke P, Javier Luque F, Murray JB, Barril X (2011) Shielded hydrogen bonds as structural determinants of binding kinetics: Application in drug design. J Am Chem Soc 133:18903–18910. 10.1021/ja207494u 21981450 10.1021/ja207494u

[CR60] Schneider NFZ, Cerella C, Simões CMO, Diederich M (2017) Anticancer and immunogenic properties of cardiac glycosides. Molecules 22. 10.3390/molecules22111932 10.3390/molecules22111932 PMC6150164 29117117

[CR61] Schneider NFZ, Persich L, Rocha SC, Ramos ACP, Cortes VF, Silva IT et al (2018) Cytotoxic and cytostatic effects of digitoxigenin monodigitoxoside (DGX) in human lung cancer cells and its link to Na,K-ATPase. Biomed Pharmacother 97:684–696. 10.1016/j.biopha.2017.10.128 29101813 10.1016/j.biopha.2017.10.128

[CR62] Slingerland M, Cerella C, Guchelaar HJ, Diederich M, Gelderblom H (2013) Cardiac glycosides in cancer therapy: From preclinical investigations towards clinical trials. Invest New Drugs 31:1087–1094. 10.1007/s10637-013-9984-1 23748872 10.1007/s10637-013-9984-1

[CR63] Smedler E, Uhlén P (2014) Frequency decoding of calcium oscillations. Biochim et Biophys Acta (BBA) - Gen Subj 1840:964–969. 10.1016/j.bbagen.2013.11.015 10.1016/j.bbagen.2013.11.015 24269537

[CR64] Stenlund P, Babcock GJ, Sodroski J, Myszka DG (2003) Capture and reconstitution of G protein-coupled receptors on a biosensor surface. Anal Biochem 316:243–250. 10.1016/S0003-2697(03)00046-0 12711346 10.1016/s0003-2697(03)00046-0

[CR65] Stolz M, Lewitzki E, Schick E, Mutz M, Grell E (2003) Calorimetry of Na,K-ATPase. New York Acad Sci 986:245–246 10.1111/j.1749-6632.2003.tb07172.x 12763808

[CR104] Taniguchi K, Post RL (1975) Synthesis of adenosine triphosphate and exchange between inorganic phosphate and adenosine triphosphate in sodium and potassium ion transport adenosine triphosphatase. Biol Chem 250(8):3010–3018 123528

[CR66] Tarcsay Á, Keserű GM (2015) Is there a link between selectivity and binding thermodynamics profiles? Drug Discov Today 20:86–94. 10.1016/j.drudis.2014.09.014 25263698 10.1016/j.drudis.2014.09.014

[CR100] Toledo MM, De Souza Gonçalves B, Colodette NM, Chaves ALF, Muniz LV, De A Ribeiro RIM (2021) Tumor tissue oxidative stress changes and Na, K-ATPase evaluation in head and neck squamous cell carcinoma. J Membr Biol. 254(5-6):475–486. 10.1007/s00232-021-00185-y 10.1007/s00232-021-00185-y 34104985

[CR67] Tverskoi АM, Poluektov YM, Klimanova EA, Mitkevich VA, Makarov AA, Orlov SN et al (2021) Depth of the steroid core location determines the mode of Na,K-ATPase inhibition by cardiotonic steroids. Int. J. Mol. Sci*.* 22. 10.3390/ijms222413268 10.3390/ijms222413268 PMC8708600 34948068

[CR68] Van’t Hoff JH (1884) Études de dynamique chimique. Frederik Muller & Co: Amsterdam

[CR69] Vauquelin G (2016) Effects of target binding kinetics on in vivo drug efficacy: koff, kon and rebinding. Br J Pharmacol 2319–2334. 10.1111/bph.13504 10.1111/bph.13504 PMC4945762 27129075

[CR101] Vauquelin G, Charlton SJ (2010) Long-lasting target binding and rebinding as mechanisms to prolong in vivo drug action. Br. J. Pharmacol. 161:488–508. 10.1111/j.1476-5381.2010.00936.x 10.1111/j.1476-5381.2010.00936.x PMC2990149 20880390

[CR200] Vauquelin G, Charlton SJ (2013) Exploring avidity: understanding the potential gains in functional affinity and target residence time of bivalent and heterobivalent ligands. Br J Pharmacol. 168:1771–1785. 10.1111/bph.12106 10.1111/bph.12106 PMC3623049 23330947

[CR70] Vieira L, Saldanha AA, Moraes AM, de Oliveira FM, Lopes DO, de Barbosa LA O., et al (2018) 21-Benzylidene digoxin, a novel digoxin hemi-synthetic derivative, presents an anti-inflammatory activity through inhibition of edema, tumour necrosis factor alpha production, inducible nitric oxide synthase expression and leucocyte migration. Int Immunopharmacol 65:174–181. 10.1016/j.intimp.2018.10.010 30316075 10.1016/j.intimp.2018.10.010

[CR71] Winquist J, Geschwindner S, Xue Y, Gustavsson L, Musil D, Deinum J et al (2013) Identification of structural-kinetic and structural-thermodynamic relationships for thrombin inhibitors. Biochemistry 52:613–626. 10.1021/bi301333z 23290007 10.1021/bi301333z

[CR120] Xu Y, Marck P, Huang M, Xie JX, Wang T, Shapiro JI, Cai L, Feng F, Xie Z (2021) Biased effect of cardiotonic steroids on Na/K-ATPase–Mediated signal transduction. Mol. Pharmacol 99(3):217–225. 10.1124/molpharm.120.000101 10.1124/molpharm.120.000101 PMC7919863 33495275

[CR72] Yang P, Cartwright C, Efuet E, Hamilton SR, Wistuba II, Menter D et al (2014) Cellular location and expression of Na+,K+-ATPase α subunits affect the anti-proliferative activity of oleandrin. Mol Carcinog 53:253–263. 10.1002/mc.21968 23073998 10.1002/mc.21968 PMC4442617

[CR73] Yatime L, Laursen M, Morth JP, Esmann M, Nissen P, Fedosova NU (2011) Structural insights into the high affinity binding of cardiotonic steroids to the Na+,K+-ATPase. J Struct Biol 174:296–306. 10.1016/j.jsb.2010.12.004 21182963 10.1016/j.jsb.2010.12.004

